# Voluntary work for the physical and mental health of older volunteers: A systematic review

**DOI:** 10.1002/cl2.1124

**Published:** 2020-10-23

**Authors:** Trine Filges, Anu Siren, Torben Fridberg, Bjørn C. V. Nielsen

**Affiliations:** ^1^ VIVE‐Campbell Copenhagen Denmark

## Abstract

**Background:**

The increasing imbalance between the number of older adults not working and the number of adults in the age range of labour force participation (age range 20–64) has long been a fundamental public policy challenge in the Organization for Economic Co‐operation and Development member countries. At a societal level, this growing imbalance raises serious concerns about the viability and funding of social security, pensions and health programmes. At an individual level, the concern is probably more that of aging well with the prospect of many years in retirement. Some research suggests that retiring for some carries the risk of a fast decline in health. Volunteering can play a significant role in people's lives as they transition from work to retirement, as it offers a “structured” means of making a meaningful contribution in society once the opportunity to do so through work has been cut off. Some older people consider voluntary work as a way to replicate aspects of paid work lost upon retirement, such as organisational structure and time discipline. In many countries, volunteering of the older adults is increasing and programmes designed specifically for this subpopulation are emerging. Volunteering may contribute to both individuals aging well and society aging well, as volunteering by the older adults at the same time relieves the societal burden if it helps maintain health and functionality for those who volunteer. It thus remains to be established to what extent volunteering impacts on the physical and mental health of those who volunteer.

**Objectives:**

The main objective of this review is to answer the following research question: what are the effects of volunteering on the physical and mental health of people aged 65 years or older?

**Search Strategy:**

Relevant studies were identified through electronic searches of bibliographic databases, governmental and grey literature repositories, hand search in specific targeted journals, citation tracking, contact to international experts and internet search engines. The database searches were carried out to December 2018 and other resources were searched in September 2019 and October 2019. We searched to identify both published and unpublished literature. The searches were international in scope. Reference lists of included studies and relevant reviews were also searched.

**Selection Criteria:**

The intervention of interest was formal volunteering which can be described as voluntary, on‐going, planned, helping behaviour that intend to increase the well‐being of strangers, offers no monetary compensation and typically occurs within an organisational context. We included older people aged 65 or over who are engaged in formal voluntary work. The primary focus was on measures of physical and mental health. All study designs that used a well‐defined control group were eligible for inclusion. Studies that utilised qualitative approaches were not included.

**Data Collection and Analysis:**

The total number of potential relevant studies constituted 17,046 hits. A total of 90 studies, met the inclusion criteria and were critically appraised by the review authors. The 90 studies analysed 47 different populations. Only 26 studies (analysing 19 different populations) could be used in the data synthesis. Forty‐six studies could not be used in the data synthesis as they were judged to have too high risk of bias and, in accordance with the protocol, were excluded from the meta‐analysis on the basis that they would be more likely to mislead than inform. Eighteen studies did not provide enough information enabling us to calculate an effects size and standard error or did not provide results in a form enabling us to use it in the data synthesis. Finally, of the 26 studies that could be used in the data synthesis, two pairs of studies used the same two data sets and reported on the same outcome(s), thus in addition two studies were not used in the data synthesis.

Meta‐analysis of both physical health outcomes and mental health outcomes were conducted on each metric separately. All analyses were inverse variance weighted using random effects statistical models that incorporate both the sampling variance and between study variance components into the study level weights. Random effects weighted mean effect sizes were calculated using 95% confidence intervals (CIs).

Sensitivity analysis was carried out by restricting the meta‐analysis to a subset of all studies included in the original meta‐analysis and was used to evaluate whether the pooled effect sizes were robust across components of risk of bias.

**Results:**

The 24 studies (analysing 19 different populations), used for meta analysis were from Australia, Ireland, Israel, Japan, Korea and United States, three were a randomised controlled trial and 21 were NRS. The baseline time period (the year the voluntary work that was analysed was measured) spanned by the included studies is 30 years, from 1984 to 2014 and on average the baseline year was 2001. On average the number of follow up years was 5, although with great variation from 0 to 25 years. The average number of volunteers analysed (not reported in four studies) was 2,369, ranging from 15 to 27,131 and the average number of controls was 13,581, ranging from 13 to 217.297. In total the average number of participants analysed was 14,566, ranging from 28 to 244.428.

Ten studies analysed the effect of voluntary work on mortality, however, eight studies reported a hazard ratio and two studies reported an odds ratio. We analysed these two types of effect sizes separately. A hazard ratio <1 indicates that the treated, the volunteers is favoured. That is, the conditional mortality rate is lower for volunteers. All reported results indicated an effect favouring the volunteers, primary study effect sizes lied in the range 0.67–0.91. The random effects weighted mean hazard ratio was 0.76 (95% CI, 0.72–0.80) and statistically significant. The two studies that reported odds ratios of mortality supported this result. There was no heterogeneity between the studies in either of the meta analyses.

Three studies analysed the effect of voluntary work on incident functional disability, using a hazard ratio as effect measure. All reported results indicated an effect favouring the volunteers, primary study effect sizes lied in the range 0.70–0.99. The random effects weighted mean hazard ratio was 0.83 (95% CI, 0.72–0.97) and statistically significant. There was a small amount of heterogeneity between the studies.

Two studies analysed the effect of voluntary work on decline in instrumental activities of daily living, using an odds ratio as effect measure. Both reported results indicated an effect favouring the volunteers (0.63 and 0.83). The random effects weighted mean odds ratio was 0.73 (95% CI, 0.53–1.01) and not statistically significant. There is no heterogeneity between the two studies.

Three studies analysed the effect of voluntary work on maintenance of functional competence, using an odds ratio as effect measure. All reported results indicated an effect favouring the volunteers, primary study effect sizes lied in the range 0.67–0.83. The random effects weighted mean odds ratio was 0.81 (95% CI, 0.70–0.94) and statistically significant. There is no heterogeneity between the studies.

In addition a number of other physical outcomes were reported in a single study only.

Three studies analysed the effect of voluntary work on depression, and reported results that enabled the calculation of standardised mean difference (SMD) and variance. The effect sizes are measured such that a positive effect size favours the volunteers. All reported results indicated an effect favouring the volunteers, primary study effect sizes lied in the range 0.05–0.66. The random effects weighted SMD was 0.12 (95% CI, 0.00–0.23) and statistically significant. There is a very small amount of heterogeneity between the studies.

In addition, a number of other mental health outcomes were reported in a single study only.

We did not find any adverse effects.

There were no appreciable changes in the results across components of risk of bias as indicated by the sensitivity analysis.

**Authors' Conclusions:**

The review aimed to examine effects on all types of physical and mental health outcomes. With the exception of mortality, there was insufficient evidence available. The available evidence, however, does suggest that there is an effect on the mortality of volunteers, although the effect is small. We found evidence that voluntary work reduces the mortality hazard of the volunteers aged 65 and above. The effect corresponds to a 43% chance of the volunteers dying first which should be compared to a fifty‐fifty chance (50%) of dying first if the intervention had no effect. The evidence seems robust in the sense that we did not find any heterogeneity between the studies. As the intervention, unlike most other interventions in the social welfare area, is not costly, it could be prescribed to more older adults. In fact as the intervention in contrary to carrying a cost is a productive activity contributing directly to community well‐being and has a positive effect on the volunteers it probably should be prescribed universally. However, due to the very nature of the intervention, it is voluntary and it cannot be prescribed. But more people could be encouraged to take up voluntary work if the opportunity was immediately available and visible.

## 1 PLAIN LANGUAGE SUMMARY

### 1.1 Volunteering improves the physical and mental health of older volunteers

There are increasing numbers of older adults who no longer work. Volunteering has the double benefit of the activity for these adults and the value of the services they provide as volunteers. The evidence suggests that volunteering improves the physical and mental health of volunteers, notably a reduction in mortality. The evidence is inconclusive for other outcomes because of the small number of studies.

### 1.2 What is this review about?

The increasing imbalance between the number of older adults not working and the number of adults in the age range of labour force participation is a fundamental public policy challenge in high‐income countries. Retiring may carry the risk of a fast decline in health.

Volunteering can play a significant role in people's lives as they transition from work to retirement. Volunteering may contribute to individuals ageing well and society ageing well because volunteering by older adults relieves a societal burden while helping these volunteers to maintain health and functionality.

This review examines the evidence of impact of volunteering on the physical and mental health of older people who volunteer.

**What is the aim of this review?**

This Campbell systematic review examines the effects volunteering on the physical and mental health of older people who volunteer. The review summarises evidence from 26 studies undertaken in Australia, Ireland, Israel, Japan, Korea and United States which involved over 47,000 volunteers in total.

### 1.3 What studies are included?

Included studies had to examine the impact of formal volunteering by people aged 65 or over on their physical and mental health. Studies had to have a comparison group.

Ninety studies analysing 47 different populations were identified. Of these, only 26 studies, analysing 19 different populations, could be used in the data synthesis. The studies were from Australia, Ireland, Israel, Japan, Korea and United States. Three were randomised controlled trials (RCTs) and 21 were nonrandomised studies. The studies contained data for over 47,000 volunteers.

What is the effect of volunteering on the physical and mental health of older people?

Volunteering improves the physical and mental health of volunteers. The effect is best documented for mortality, with too few studies to draw conclusions for other outcomes.

For physical health, the evidence shows that there is a positive effect on reducing mortality (10 studies) and possibly incident functional disability (three studies). Volunteering may support the maintenance of functional competence (three studies) and slow the decline in instrumental activities of daily living (IADL; two studies).

For mental health, volunteering may reduce depression (three studies).

There was no evidence of adverse effects.

In all cases, there is little variation in the estimated effects from the different studies. However, the effects are small, and except in the case of mortality, based on a small number of studies.

### 1.4 What do the findings of the review mean?

Voluntary work reduces the mortality hazard of volunteers aged 65 and above. The effect corresponds to a 43% chance of the volunteers dying first which should be compared to a fifty‐fifty chance (50%) of dying first if the intervention had no effect. The evidence seems robust as there is little variation in findings between the studies.

As the intervention—unlike most other interventions in the social welfare area—is not costly, it could be prescribed to more older adults. In fact, contrary to carrying a cost, volunteering is a productive activity contributing directly to community well‐being.

As volunteering has a positive effect on the volunteers, it probably should be prescribed universally. Given the very nature of the intervention, however, it is voluntary and cannot be prescribed. Nevertheless, measures can be taken to encourage people to engage in voluntary work by making opportunities more immediately available and visible.

### 1.5 How up‐to‐date is this review?

The review authors searched for studies published up to October 2019.

## 2 BACKGROUND

### 2.1 The problem, condition or issue

The increasing imbalance between the number of older adults not working and the number of adults in the age range of labour force participation (age range 20–64) has long been a fundamental public policy challenge in the Organization for Economic Co‐operation and Development (OECD) member countries^1^ (OECD, 2015). The large cohorts born after World War II (WWII), born appr. from 1946 to 1955, grow older and as a consequence, social scientists and policy makers have taken an intense interest in how their aging and eventual retirement from the full‐time labour force will affect society.

In only 15 years, the share of the population aged 65 and over in the OECD countries has increased by more than 4% points; from 13% in 2000 to more than 17% in 2018 (OECD. Stat, data extracted April 3, 2020). The effect of an aging population on a country's societal support burden is often measured by the older dependency ratio, which is the ratio of the older population to the working‐age population. The OECD average older dependency ratio (ratio of individuals aged 65 and above to those aged 15–64) has increased considerably over the last half century, from 15.2 in 1970 to 26.4 in 2018 (OECD. Stat, data extracted April 3, 2020). The problem is more pronounced in Europe than in the United States; the older dependency ratio was 24.5 in the United States in 2018 and as high as 30.7 in Europe^2^ in 2018 (OECD. Stat, data extracted April 3, 2020).

In addition to the large post‐WWII cohorts growing older, the average duration of expected years in retirement has increased. In 1970, men in the OECD countries spent on average 11 years in retirement and by 2014, this average had increased to almost 18 years (OECD, 2014). The increase for women has been from 15 years in 1970 to 22.3 years in 2014.

The increase in average duration of years in retirement is partly due to increased longevity and partly due to earlier retirement. Although the effective age of retirement (the average effective age at which workers withdraw from the labour force) has slowly started to increase since 2004, it decreased steadily for 30 years between 1970 and 2001 (OECD, 2014). In 2018, the effective retirement age was on average 64.2 for men (63.5 for women) in the OECD countries; somewhat higher in the United States (66.0 for both men and women) than in Europe^3^ (64.2 for men and 63.3 for women) (OECD. Stat, data extracted April 3, 2020).

By 2050, the population aged 65 and over in the United States is expected to grow to almost 21% and the older dependency ratio is estimated to increase to 38 (OECD. Stat, data extracted August 22). In Europe, the percentage of the population aged 65 and over is expected to grow to almost 29% by 2050, and the older dependency ratio is estimated to increase to 55 (OECD. Stat, data extracted August 22). At a societal level, this growing imbalance raises serious concerns about the viability and funding of social security, pensions, and health programmes.

At an individual level, the concern is probably more that of aging well with the prospect of many years in retirement. Some research suggests that retiring for some carries the risk of a fast decline in health (Dave et al., 2008; Szinovacz & Davey, 2004). Evidence on whether, and if so, how retirement influences health and wellbeing is however inconclusive (Biggs et al., 2017). The effects of retirement have been found to depend, for example, on the type of work (e.g., Vickerstaff, 2010) and whether the retirement is forced or voluntary (Hershey & Henkens, 2013). Nevertheless, for many people, work can provide meaningful roles, social contacts and structured everyday life experience (Jahoda, 1981, 1982, 1984; Seeman, 1996; Warr, 1994). Thus, loosing these aspects in one's life when transitioning to retirement can have negative implications for wellbeing and health of individuals. Several studies have demonstrated that subjective usefulness is strongly related to both physical and psychological health (Ranzijn et al., 1998; Ryan & Frederick, 1997; Ryff, 1989). The performance of other meaningful (for the individual) activities than working for pay may thus help maintain health and functional ability for older people.

Using U.S. data from 1995 and 2005, Einolf (2009) predicted that post‐WWII cohorts' rate of volunteering at the time of their retirement will be higher than that of earlier cohorts. Given the large size of these post‐WWII cohorts, Einolf concluded that the total number of older volunteers would increase. The prediction seems to hold true, at least for those countries where it has been possible to locate relevant numbers for the baby boom generation's rate of volunteering. In Canada, the rate of volunteering for those aged 65 and over increased from 32% in 2004 to 36% in 2010 (Vézina & Crompton, 2012), and in Denmark the rate increased from 23% in 2004 to 34% in 2012 (Fridberg & Henriksen, 2014). A recent study in Denmark showed that while the rates of volunteering remained stable for younger seniors, there was a large increase over time among those aged 67–77 years (Amilon & Larsen, 2020).

Researchers have partly explained the increasing in the rates of older volunteers by increasing heath and active ageing lifestyles among older adults (e.g., Amilon & Larsen, 2020; Schippers & Conen, 2014). Partly the development has been explained by volunteer work becoming increasingly professionalized and the recruitment strategies to integrate older people in the volunteer organisations being increasingly efficient (Amilon & Larsen, 2020). Many countries in Europe have explicit strategies to increase older adults' volunteering (Ehlers, Naegele, & Reichert, 2011). In the United States, specific programmes aiming to integrate the aging population into voluntary work exist. Some programmes are organised in local nonprofit organisations, referred to as Senior Corps Programs, a network of national service programmes that provides the opportunity for people aged 55 years or above to apply their life experience to meeting community needs (see www.seniorcorps.org/rsvp/senior-corps-programs-2/).

### 2.2 The intervention

Volunteering is a complex phenomenon and spans a wide variety of types of activities, organisations and sectors. The intervention of interest in this review is formal volunteering. Formal volunteering can be described as voluntary, on‐going, planned, helping behaviour that intend to increase the well‐being of strangers, offers no monetary compensation and typically occurs within an organisational context (Clary et al., 1998; Penner, 2002). We will define formal volunteering centred on four axes (as defined in Hustinx et al., 2010). These are:

(a) Free will: volunteering is a free choice; it is a voluntary action as opposed to a compulsory action. (b) Remuneration: the voluntary work offers no monetary compensation. There may be reimbursement for expenses incurred but otherwise the work is unpaid. (c) Intended beneficiaries: volunteer work can be described as “unpaid work provided to parties to whom the worker owes no contractual, familial or friendship obligations” (Tilly & Tilly, 1994, p. 291). Thus, formal volunteer work typically benefits strangers and is often referred to as nonobligatory helping (Omoto & Snyder, 1995). (d) Structure: volunteering as defined here should involve planned and ongoing activities (as opposed to a spontaneous one‐time activity). Such planned and ongoing activities often occur in some type of organisational context (Penner, 2002). An organisation defines the content of the volunteer work and formulates some expectations to the volunteer, including the tasks of the volunteer worker. The organisation produces plans, recruits the volunteers, educates them if necessary, and leads them. Thus, the relations that occur in the voluntary work are formal and different from the informal relations that are found between friends and family members to whom the volunteer may feel obliged (La Cour, 2014).

### 2.3 How the intervention might work

Volunteering can play a significant role in people's lives as they transition from work to retirement. According to Smith and Gay (2005), retirement is a trigger for volunteering for some older people, as it offers a “structured” means of making a meaningful contribution in society once the opportunity to do so through work has been cut off. Some older people consider voluntary work as a way to replicate aspects of paid work lost upon retirement, such as organisational structure and time discipline (Smith & Gay, 2005). The same line of arguments for volunteering can be found in several other studies (see, Chappell & Prince, 1997; Fischer et al., 1991; Greenfield & Marks, 2004; Newman et al., 1985; Widjaja, 2010). Volunteering thus seems to provide a way of compensating for the losses due to retirement as identified by Jahoda (1981, 1982, 1984), such as the need for time structure, social contact, collective effort or purpose, social identity or status, and regular activity. Several studies argue that there is a potential health benefit to older volunteers and in particular retirees (Moen & Fields, 2002; Musick & Wilson, 2003; Young & Glasgow, 1998).

**Figure 1:**
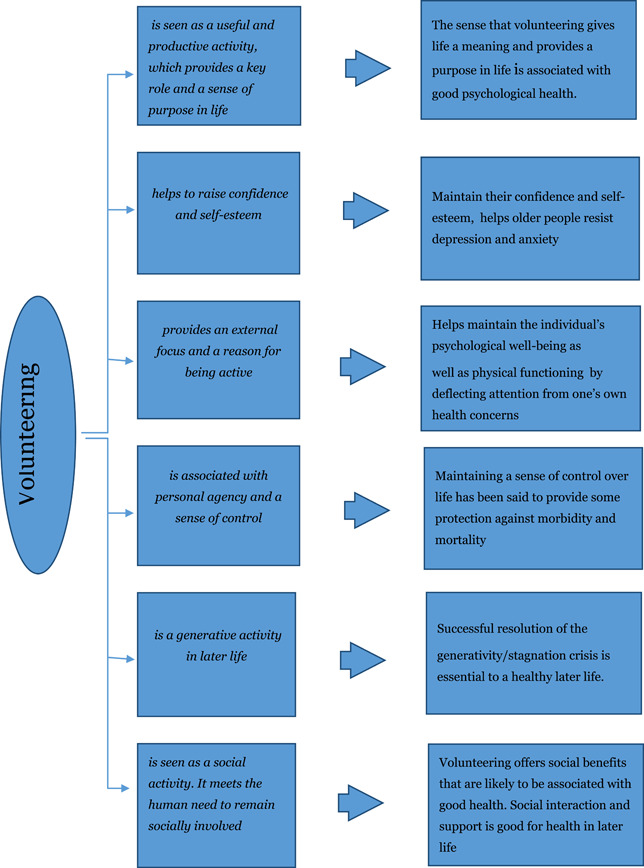
Mechanisms by which volunteering may affect health

The exact mechanisms and processes linking volunteering and health for older people has however not been sufficiently explored and may be very complex (Warburton, 2006). Using a qualitative approach Warburton (2006) explored this relationship and identified six potential themes and the impacts on health is discussed. The six themes and their impacts on health are illustrated in Figure 1.

### 2.4 Why it is important to do the review

In many countries, volunteering of the older adults is increasing and programmes designed specifically for this subpopulation are emerging. Volunteering may contribute to both individuals aging well and society aging well, as volunteering by the older adults at the same time relieves the societal burden if it helps maintain health and functionality for those who volunteer. It thus remains to be established to what extent volunteering impacts on the physical and mental health of those who volunteer.

Health status is often found to be an important predictor of volunteering among those aged 65 years or more, see, for example, Brown (2000) and Young and Glasgow (1998). The question that is important to answer is: does good health predict volunteering or does volunteering improve health (or maybe both)? Studies that simply assess the association between voluntary work and health outcomes cannot answer this question. Research using appropriate controls and outcome measures can, however, provide some relevant evidence on whether engaging in voluntary work might cause good health outcomes on older people. It is vital that an appropriate comparison group is used to establish the direction of cause. Does volunteering make people healthier, or are healthier people more likely to volunteer? Likewise, it is vital that the health measures are objective. As stated in Wilson and Musizk (1999, p. 153): “[C]ross‐sectional designs that use participants to self‐assess the impact of a volunteer program function as little more than market research for the agency concerned. Without a pre/posttest design and a control group, and without more objective and generalisable outcome measures, little can be learned of the benefits of volunteering from these studies”. The same worries concerning reliance on cross‐sectional designs and self‐assessment of health to establish causality can be found in Lum and Lightfood (2005). Hence, considering the fact that the population under investigation in this review by nature volunteer into the intervention, we believe it is vital that an appropriate comparison group and access to relevant pre health measures and objective health measures are used to establish causality.

We are very clear that firm causal conclusions probably cannot be drawn from the studies we included in the review, as we found only a few studies based on randomised trials. However, a distinction can be drawn between studies that simply assess the association between voluntary work and health outcomes, and studies that control for important confounding factors, in particular pre health measures, and use objective health measures. Studies that control for important confounding factors and use objective health measures provide some evidence for considering possible causal effects. While conclusions about causal effects must be very tentative, it is important to extract and summarise the best evidence available.

An obvious question arises: is there any value in conducting a systematic review when it is likely that there are no trial based studies available? We think it is worthwhile as a systematic review may uncover high quality studies that may not be found using less thorough searching methods. Furthermore, if a systematic review demonstrates that high quality studies are lacking, this could encourage a new generation of primary research. Therefore, even though we did not expect to find any trial based studies (and only found a few) and only a limited number of studies of voluntary work based on appropriate outcome measures and control group comparison, we still believe there is value in conducting a review in order to gather and highlight the best available knowledge.

## 3 OBJECTIVES

The main objective of this review is to answer the following research question: what are the effects of volunteering on the physical and mental health of people aged 65 years or older?

## 4 METHODOLOGY

The systematic review protocol was published on November 27, 2018. The protocol is available at https://onlinelibrary.wiley.com/doi/10.1002/CL2.190.

### 4.1 Criteria for including and excluding studies

#### 4.1.1 Types of study designs

It is hard to imagine that a researcher would randomise the allocation of people to volunteer work. We therefore anticipated that relatively few RCTs on the effects of volunteer work on the health of the volunteers would be found. However, we found a few and they were of course included in the review. In order to summarise what is known about the possible causal effects of volunteering, we included all study designs that use a well‐defined control group. Nonrandomised studies, where voluntary work has occurred in the course of usual decisions outside the researcher's control, must have demonstrated pretreatment group equivalence via matching, statistical controls or evidence of equivalence on key risk variables and participant characteristics. These factors are outlined in Section 4.5.2, and the methodological appropriateness of the included studies were assessed according to the risk of bias model.

The study designs eligible for inclusion in the review were:

A. RCTs (where all parts of the study are prospective, such as identification of participants, assessment of baseline, and allocation to intervention, and which may be randomised, quasi randomised or nonrandomised), assessment of outcomes and generation of hypotheses (Higgins & Green, 2008).
B. Nonrandomised studies (voluntary work has occurred in the course of usual decisions, the allocation to working voluntary and not working voluntary is not controlled by the researcher, and there is a comparison of *two or more groups* of participants).

#### 4.1.2 Types of participants

The “intervention population” were people aged 65 or over who are engaged in formal voluntary work. Studies where the majority of participants were aged 65 or over, or where results were shown for subgroups of participants aged 65 or over, were included. We included voluntary workers of both genders and all nationalities who performed all types of formal voluntary work as defined in the Intervention section.

#### 4.1.3 Types of interventions

The intervention of interest in this review is formal volunteering. Formal volunteering can be described as voluntary, on‐going, planned, helping behaviour that intend to increase the well‐being of strangers, offers no monetary compensation and typically occurs within an organisational context (see Section 2). Informal ways of helping friends, neighbours or relatives, such as running errands, providing transportation and so forth, which are typically motivated by an obligation to help intimate others, were excluded.

The comparison population were people who are not engaged in formal voluntary work.

#### 4.1.4 Types of outcome measures

The primary focus was on measures of health.

#### 4.1.5 Primary outcomes

As primary outcomes we planned to include physical health outcomes as well as mental health outcomes. All measures of physical health outcomes reported in studies using a comparable control group had to be objective in order to be included as primary outcomes. As mentioned above Wilson and Musizk (1999) highlight the problem with studies relying on self‐assessment of the impact of volunteering. Self‐assessment of health should not be confused with self‐reported measures. By self‐assessment we understand questions of the form: “Would you say the state of your health is excellent, good, fair, poor or very poor?” which was not included as a primary outcome. On the other hand, we did not expect that measures of mental health outcomes be obtained via structured clinical interviews. Instead, we expected that self‐reported questionnaires be used to screen for probable mental disorders.

The use of different instruments of detection may be an important source of variation for the incidence of measured mental health outcomes. Measures of health had to be standardised to be included, see below. General scales of well‐being were included if they were measured by standardised psychological symptom measures.

Standardised physical health outcomes reported in the included studies were mortality, physical activity (measured by the modified Minnesota Leisure Time Physical Activity Questionnaire), incident functional disability (defined as certification for LTCI, a mandatory form of social insurance to assist the daily activity of frail older individuals in Japan), decline in IADL, cognitive impairment (using the Mini‐Mental State Examination), maintenance of functional health (measured by the Tokyo Metropolitan Institute of Gerontology Index of Competence), incident cardiovascular disease (CVD; nonfatal heart attack or stroke, or a CVD‐related death, all in one measure), functional impairment (measured using a combination of IADL and Activities of daily living (ADL)), number of doctor visits and number of nights in hospital.

Standardised mental health outcomes reported in the included studies were loneliness (measured by the three‐item UCLA Loneliness scale), psychological well‐being (measured by the Warwick–Edinburgh Mental Well‐Being Scale), depression (measured by the Center for Epidemiological Studies Depression Scale and the Geriatric Depression Scale), self‐efficacy (measured by the General Self‐Efficacy Scale), quality of life (measured by the Global Quality of Life Scale), purpose of life (measured by the Purpose in Life and Personal Growth subscales of Ryff's Psychological Well‐Being Scales), personal growth (measured by the Purpose in Life and Personal Growth subscales of Ryff's Psychological Well‐Being Scales), self‐esteem (measured by the Rosenberg Self‐Esteem Scale) and life satisfaction (measured by the Life Satisfaction Index–A).

#### 4.1.6 Secondary outcomes

Although some researchers express concerns about using self‐assessed health measures (Lum & Lightfood, 2005; Wilson & Musizk, 1999), others argue that self‐assessed health can be a good predictor of mortality (Jylhä, 2009; Jylhä et al., 2006). If studies reported self‐assessment of health, using questions of the form: “Would you say the state of your health is excellent, good, fair, poor or very poor?” they were included as secondary outcomes.

Nonstandardised physical and mental health outcomes reported in the included studies were decline in cognitive performance, self‐rated health, functional limitations and life satisfaction.

#### 4.1.7 Duration of follow‐up

Time points planned for measures were:

- While actively engaged in voluntary work
- At cessation of volunteering to 1 year after cessation of volunteering
- More than 1 year after cessation of volunteering

There were three RCT's used in the meta analysis, with measures taken at post intervention and one of these RCT's in addition reported on a 3‐month follow‐up. The remaining studies used in the meta analyses reported the year where the baseline measure of doing volunteer work was taken from and the number of years the participants were followed. However, whether the participants continued working as a volunteer throughout the follow up period or stopped at some time during the follow up period was not reported (with one exception).

#### 4.1.8 Types of settings

We planned to include volunteer work done in all organisational contexts such as religious organisations, educational organisations, health organisations political groups, sports clubs, cultural organisations, senior citizen groups or related organisations. One study used in the meta analysis reported type of voluntary work (education), the remaining studies did not explicitly report what type of organisation the work was done for.

### 4.2 Search strategy

The search was performed by one review author (B. C. N.) and two members of the review team (CAKI and ANDA).^4^

Relevant studies were identified through searches in electronic databases, governmental and grey literature repositories, hand search in specific targeted journals, citation tracking, contact to international experts and internet search engines.

#### 4.2.1 Electronic databases

Following databases were searched:

- SocIndex (EBSCO)
- PsycInfo (EBSCO)
- EconLit (EBSCO)
- Academic Search (EBSCO)
- Science Citation Index (Web of Science)
- Social Science Citation Index (Web of Science)
- MEDLINE (PubMed)
- Social Care Online

The database searches were performed between November 6, 2018 and the December 12, 2018.

#### 4.2.2 Description of the search string

The search string is based on the PICO(s)‐model, and contains three concepts, of which we have developed three corresponding search facets for the population, the intervention and outcomes. The reasoning for not developing a search facet for study type or methodology, that in general would ensure a higher level of precision, were based on a concern of excluding potential relevant references. The subject terms in the facets was chosen accordingly to the options on each separate database.

A few modifications where applied to the search as it is described in the protocol. The intended purpose of the facet for voluntary activities was not sufficiently limited by searching exclusively on terms for volunteering (volunt*), which in some cases resulted in an unreasonably high recall. In order to increase the precision of the facet, we implemented an additional set of terms covering activities, such as engagement, work, participation or involvement that was combined with the volunteering terms. The modifications does not change results of the searches in a way that differ from the intended purpose of the search strategy.

Furthermore, an additional search for named programmed interventions, such as “Senior Corps”, “Senior Companion Program”, “Foster Grandparents Program”, “Retired Senior and Volunteer Program” and “Experience Corps”.

#### 4.2.3 Limitations of the search‐string

No year or language restrictions were implemented in the database searches.

#### 4.2.4 Hand‐search

Five specific journals were hand‐searched in the time period between 2017 and September 2019:

- The Journals of Gerontology
- American Journal of Public Health
- Gerontologist
- International Journal of Geriatric Psychiatry
- Journal of Applied Gerontology

### 4.3 Searching other resources and unpublished literature

The searches on other resources and for unpublished literature was done between the September 14, 2019 and October 9, 2019. Terms used to search others resources were based on the general search strategy. Combinations of terms such as volunteering with terms for the population (i.e., old or elderly) or the outcome terms (i.e., health outcomes) were utilised. As example of these searches can be seen in the Supporting Information Appendix under the Google Scholar search strings.

Most of the resources searched for unpublished literature includes multiple types of references. As an example, the resources listed to identify reports from national bibliographical resources also include working papers and dissertations, as well as peer‐reviewed references. The same is true for Google Scholar. In the following documentation, we have split the resources for each type of unpublished literature. The resources are listed under the category of literature we expect to be most prevalent in the resource, even though multiple types of unpublished/published literature might be identified in the resource.

#### 4.3.1 Search for working papers and conference proceedings

We searched the following resources for working papers:

- NBER Working Papers—https://www.nber.org/papers.html
- OpenGrey—http://www.opengrey.eu/
- Google Scholar—https://scholar.google.com/

#### 4.3.2 Search for reports and non‐U.S. literature

- Danish Institute for Voluntary Effort—https://frivillighed.dk/
- Danish National Research Database—http://www.forskningsdatabasen.dk/en
- UK Institute for Volunteering Research—https://www.ncvo.org.uk/institute-for-volunteering-research
- Volunteer Bénévoles/Canada—https://volunteer.ca/research-resources
- Royal Voluntary Service—https://www.royalvoluntaryservice.org.uk/
- Corporation for National and Community Service—https://www.nationalservice.gov/
- Google—https://www.google.com/

#### 4.3.3 Citation tracking

In order to identify both published studies and grey literature we utilised citation‐tracking/snowballing strategies. Our primary strategy was to citation‐track related systematic‐reviews and meta‐analyses. The review team also checked reference lists of included primary studies for new leads.

#### 4.3.4 Contact to international experts

We contacted international experts to identify unpublished and ongoing studies.

### 4.4 Criteria for determination of independent findings

To account for possible statistical dependencies, we examined a number of issues: whether individuals had undergone multiple interventions, whether there were multiple treatment groups and whether several studies were based on the same data source.

#### 4.4.1 Multiple interventions groups and multiple interventions per individuals

Studies with multiple intervention groups with different individuals were included in this review, although only intervention and control groups that meet the eligibility criteria were used in the data synthesis. To avoid problems with dependence between effect sizes we planned to apply robust standard errors (Hedges et al., 2010) and use the small sample adjustment to the estimator itself (Tipton, 2015). We used the results in Tanner‐Smith and Tipton (2014) and Tipton (2015) to evaluate if there were enough studies for this method to consistently estimate the standard errors (which there was not). See Section 4.5.4 below for more details about the data synthesis.

As there were not enough studies, we used a synthetic effect size (the average) in order to avoid dependence between effect sizes. This method provides an unbiased estimate of the mean effect size parameter but overestimates the standard error. Random effects models applied when synthetic effect sizes are involved actually perform better in terms of standard errors than do fixed effects models (Hedges, 2007a). However, tests of heterogeneity when synthetic effect sizes are included are rejected less often than nominal.

There were no studies with multiple interventions per individual.

#### 4.4.2 Multiple studies using the same sample of data

Sixteen studies analysed data from the Health and Retirement Study (HRS); three studies reported on a pilot randomised trial in Baltimore, Maryland (the experience corps); eight studies used data from Americas Changing Lives survey (ACL); four studies used data from the Asset and Health Dynamics Among the Oldest Old Study (AHEAD); two studies used data from the Personality and Total Health (PATH) Through Life Project; six studies used data collected among older adults living in Nara Prefecture, Japan; two studies used data from the Taiwan Longitudinal Study on Aging (TLSA); two studies used the Survey of Health, Ageing and Retirement in Europe (SHARE); two studies used the English Longitudinal Study of Ageing (ELSA) data; two studies used data from the Wisconsin Longitudinal Study (WLS); two studies used the Longitudinal Study on Aging (LSOA) data; two studies used the MacArthur Study of Successful Aging (MSSA) data and two studies (by the same team of authors) used data of 5401 community‐dwelling older adults aged 65 years or older who were living in Kami town, Hyogo prefecture, Japan.

We reviewed all studies, but in the meta‐analyses, we only included one estimate of the effect on a particular outcome from each sample of data in order to avoid dependencies between the “observations” (i.e., the estimates of the effect) in the meta‐analyses. The choice of which estimate to include was based on our risk of bias assessment of the studies. We chose the estimate from the study that we judged to have the least risk of bias (primarily, confounding bias).

#### 4.4.3 Multiple time points

One study reported results at both post intervention and follow up. We used the time point most comparable with the other studies.

### 4.5 Details of study coding categories

#### 4.5.1 Selection of studies and data extraction

Under the supervision of review authors, two review team assistants first independently screened titles and abstracts to exclude studies that were clearly irrelevant. Studies considered eligible by at least one assistant or studies were there is insufficient information in the title and abstract to judge eligibility, were retrieved in full text. The full texts were then screened independently by two review team assistants under the supervision of the review authors. Any disagreement of eligibility was resolved by the review authors. Exclusion reasons for studies that otherwise might be expected to be eligible were documented and presented in section *Characteristics of excluded studies*. The study inclusion criteria were piloted by the review authors (see the Supporting Information Appendix). The overall search and screening process was illustrated in a flow‐diagram. None of the review authors were blind to the authors, institutions, or the journals responsible for the publication of the articles

Two review authors independently coded and extracted data from included studies.

A coding sheet was piloted on several studies and revised as necessary (see the Supporting Information Appendix). Disagreements were minor and were resolved by discussion. Data and information was extracted on: available characteristics of participants, intervention characteristics and control conditions, research design, sample size, risk of bias and potential confounding factors, outcomes and results. Extracted data was stored electronically. Analysis was conducted in RevMan5.

#### 4.5.2 Assessment of risk of bias in included studies

We assessed the risk of bias using a model developed by Prof. Barnaby Reeves in association with the Cochrane Non‐Randomised Studies Methods Group.^5^ This model is an extension of the Cochrane Collaboration's risk of bias tool and covers risk of bias in nonrandomised studies that have a well‐defined control group.

The extended model is organised and follows the same steps as the risk of bias model according to the 2008‐version of the Cochrane Hand book, chapter 8 (Higgins & Green, 2008). The extension to the model is explained in the three following points:

1 The extended model specifically incorporates a formalised and structured approach for the assessment of selection bias in nonrandomised studies by adding an explicit item that focuses on confounding.^6^ This is based on a list of confounders considered important and defined in the protocol for the review. The assessment of confounding is made using a worksheet, which is marked for each confounder according to whether it was considered by the researchers, the precision with which it was measured, the imbalance between groups, and the care with which adjustment was carried out (see the Supporting Information Appendix). This assessment informs the final risk of bias score for confounding.
2 Another feature of nonrandomised studies that make them at high risk of bias is that they need not have a protocol in advance of starting the recruitment process. The item concerning selective reporting therefore also requires assessment of the extent to which analyses (and potentially, other choices) could have been manipulated to bias the findings reported, for example, choice of method of model fitting, potential confounders considered/included. In addition, the model includes two separate yes/no items asking reviewers whether they think the researchers had a prespecified protocol and analysis plan.
3 Finally, the risk of bias assessment is refined, making it possible to discriminate between studies with varying degrees of risk. This refinement is achieved by the use of a 5‐point scale for certain items (see the following section *Risk of bias judgement items* for details).

The refined assessment is pertinent when considering data synthesis as it operationalizes the identification of those studies with a very high risk of bias (especially in relation to nonrandomised studies). The refinement increases transparency in assessment judgements and provides justification for excluding a study with a very high risk of bias from the data synthesis.

##### Risk of bias judgement items

The risk of bias model used in this review is based on nine items (see the Supporting Information Appendix).

The nine items refer to:

- **Sequence generation** (judged on a low/high risk/unclear scale)
- **Allocation concealment** (judged on a low/high risk/unclear scale)
- **Confounders** (judged on a 5 point scale/unclear)
- **Blinding** (judged on a 5 point scale/unclear)
- **Incomplete outcome data** (judged on a 5 point scale/unclear)
- **Selective outcome reporting** (judged on a 5 point scale/unclear)
- **Other potential threats to validity** (judged on a 5 point scale/unclear)
- **A priori protocol** (judged on a yes/no/unclear scale)
- **A priori analysis plan** (judged on a yes/no/unclear scale)

In the 5‐point scale, 1 corresponds to Low risk of bias and 5 corresponds to High risk of bias. A score of 5 on any of the items assessed on the 5‐point scale translates to a risk of bias so high that the findings will not be considered in the data synthesis (because they are more likely to mislead than inform).

##### Confounding

An important part of the risk of bias assessment of nonrandomised studies is consideration of how the studies deal with confounding factors (see the Supporting Information Appendix). Selection bias is understood as systematic baseline differences between groups which can therefore compromise comparability between groups. Baseline differences can be observable (e.g., age and gender) and unobservable (to the researcher; e.g., motivation and “ability”). There is no single nonrandomised study design that always solves the selection problem. Different designs represent different approaches to dealing with selection problems under different assumptions, and consequently require different types of data. There can be particularly great variations in how different designs deal with selection on unobservables. The “adequate” method depends on the model generating participation, that is, assumptions about the nature of the process by which participants are selected into a programme. A major difficulty in estimating causal effects of voluntary work is the potential endogeneity of the individual's health condition that leads to the decision to the volunteer and if not accounted for it will yield biased estimates.

As there is no universal correct way to construct counterfactuals for nonrandomised designs, we will look for evidence that identification is achieved, and that the authors of the primary studies justify their choice of method in a convincing manner by discussing the assumption(s) leading to identification (the assumption(s) that make it possible to identify the counterfactual). Preferably, the authors should make an effort to justify their choice of method and convince the reader that the only difference between an individual who volunteers and an individual who do not volunteer is not endogenous to the individuals' health conditions. The judgement is reflected in the assessment of the confounder unobservables in the list of confounders considered important at the outset (see the Supporting Information Appendix).

In addition to unobservables, we identified the following observable confounding factors to be most relevant: age, gender, socioeconomic status, physical health and mental health. In each study, we assessed whether these factors had been considered, and in addition we assessed other factors likely to be a source of confounding within the individual included studies.

##### Importance of prespecified confounding factors

The motivation for focusing on age, gender, socioeconomic status, physical health and mental health is given below.

As age in itself is related to increased health problems, it is important that the comparison group is of same age as the volunteer group.

Socioeconomic status (e.g., education or income) is one of the strongest determinants of selection into voluntary work (Herzog & Morgan,^1993^) and in addition numerous studies of mortality have shown that mortality and health are also strongly related with socioeconomic status (Cutler & Lleras‐Muney, 2006; Lantz et al., 1998; Sorlie et al., 1995). Women have lower mortality than men (Sorlie et al., 1995) and the socioeconomic differentials are larger for women than for men (Lantz et al., 1998). Thus, gender in itself is an important confounder.

Health is the outcome of the review and as health status (physical as well as mental) often is found to be an important predictor of volunteering among those aged 65 years or more (Wilson, 2000), it is vital that the studies demonstrate pretreatment group equivalence on physical and mental health.

##### Assessment

Two review authors independently assessed the risk of bias for each included study. There were only minor disagreements and they were solved by discussion. We report the risk of bias assessment in risk of bias tables for each included study in the Supporting Information Appendix.

#### 4.5.3 Measures of treatment effect

##### Continuous outcomes

All mental health outcomes were continuous measures and effect sizes with 95% confidence intervals (CIs) were calculated, where means and standard deviations were available, or alternatively from mean differences and standard deviations (whichever were available), using the methods suggested by Lipsey and Wilson (2001). Hedges' *g* was used for estimating standardised mean differences (SMDs).

##### Dichotomous outcomes

For the majority of physical outcomes, effects were measured as either a hazard ratio with 95% CIs or as an odds ratio with 95% CIs.

The hazard ratio measures the proportional change in hazard rates between individuals who volunteer and individuals who do not volunteer. The hazard rate is defined as the event rate (in the present context, the event is, e.g., death or functional disability) at time *t* conditional on survival (no event) until time *t* or later.

The odds ratio measures the ratio of odds between individuals who volunteer and individuals who do not volunteer. Odds are the ratio of the probability of an event (e.g., death or functional disability) occurring in a group, divided by the probability of that event not occurring.

We separately pooled studies where outcomes were measured as hazard ratios and odds ratios. We performed the meta‐analyses using the log hazard ratio (log odds ratio) and variance.

#### 4.5.4 Statistical procedures and conventions

The project followed standard procedures for conducting systematic reviews using meta‐analysis techniques.

All analyses were inverse variance weighted using random effects statistical models that incorporate both the sampling variance and between study variance components into the study level weights. Random effects weighted mean effect sizes were calculated using 95% CIs.

Meta‐analysis of both physical health outcomes and mental health outcomes were conducted on each metric (as outlined in section “Types of outcomes measures”) separately.

When the effect sizes used in the data synthesis were odds ratios or hazard ratios, they were log transformed before being analysed.

Studies that were coded with a very high risk of bias (scored 5 on the risk of bias scale) were not included in the data synthesis.

We provided a graphical display (Forest plot) of effect sizes. Graphical displays for meta‐analysis performed on ratio scales sometimes use a log scale, as the CIs then appear symmetric. This is however not the case for the software Revman 5 which we used in this review. The graphical displays using odds ratios and hazard ratios and the mean effect sizes were reported as a odds ratio and a hazard ratio. Heterogeneity among primary outcome studies was assessed with *χ* ^2^ (*Q*) test, and the *I* ^2^ and *τ* ^2^ statistics (Higgins et al., 2003). Any interpretation of the *χ* ^2^ test was made cautiously on account of its low statistical power.

Several studies used the same sample of data. We reviewed all such studies, but in the meta‐analysis we only included one estimate of the effect from each sample of data. This was done to avoid dependencies between the “observations” (i.e., the estimates of the effect) in the meta‐analysis. The choice of which estimate to include was based on our quality assessment of the studies. We chose the estimate from the study that we judged to have the least risk of bias, with particular attention paid to confounding bias.

Several studies provided results separated by activity level and/or gender. There was not a sufficient number of studies included in any of the meta analyses to use robust variance estimation as planned. We conducted the meta analyses using a synthetic effect size (the average) in order to avoid dependence between effect sizes.

### 4.6 Sensitivity analysis

Sensitivity analysis was carried out by restricting the meta‐analysis to a subset of all studies included in the original meta‐analysis and was used to evaluate whether the pooled effect sizes were robust across components of risk of bias.

For methodological quality, we performed sensitivity analysis for the incomplete outcome data, the other bias and the confounding item of the risk of bias checklists, respectively. For the remaining components of the risk of bias checklist, there were no variation in the scores given.

### 4.7 Assessment of reporting bias

Reporting bias refers to both publication bias and selective reporting of outcome data and results. Here, we state how we assessed publication bias.

We used funnel plots for information about possible publication bias.

## 5 RESULTS

### 5.1 Description of studies

#### 5.1.1 Results of the search

The results are summarised in a flow chart in the Supporting Information Appendix. The total number of potential relevant records was 17,046 after excluding duplicates (database: 15,537, grey, hand search, snowballing and other resources: 1,509). All records were screened based on title and abstract; 16,674 were excluded for not fulfilling the screening criteria, two records were unobtainable despite efforts to locate them through libraries and searches on the internet and 370 records were ordered, retrieved and screened in full text. Of these, 265 did not fulfil the screening criteria and were excluded. If there was not enough information provided to to determine if the study participants met the age criteria the study authors were contacted and asked to provide the nessessary information. Fifteen studies are still awaiting classification due to uncertainty of whether the study participants meet the age eligibility criteria of the review. We could not locate a valid e‐mail address for the authors of five of these studies and we are still awaiting answers from the study authors with regard to 10 studies. A total of 90 studies were included in the review. The references are listed in section *References to included studies*.

#### 5.1.2 Included studies

The search resulted in a final selection of 90 studies, which met the inclusion criteria for this review. The 90 studies analysed 47 different populations. Only 26 studies (analysing 19 different populations) could be used in the data synthesis. Forty‐six studies could not be used in the data synthesis as they were judged to have too high risk of bias. Eighteen studies did not provide enough information enabling us to calculate an effects size and standard error or did not provide results in a form enabling us to use it in the data synthesis. Finally, of the 26 studies that could be used in the data synthesis, two pairs of studies used the same two data sets and reported on the same outcome(s), thus in addition two studies were not used in the data synthesis, see below.

Sixteen studies analysed data from the HRS; a U.S. panel survey based on a national probability sample of adults age 51 and older with each wave of data collected roughly every 2 years. Seven of these studies were not used in the data synthesis as they were judged to have a score of 5 on the risk of bias scale, four of these studies did not report data that enabled calculation of an effect size and standard error, so only five of these studies (reporting different outcomes) were used in the data synthesis.

Three studies reported on a pilot randomised trial in Baltimore, Maryland (the experience corps). One study was used in the data synthesis as it was judged to have a score of 5 on the risk of bias scale and among the two remaining studies (reporting the same outcome) we used the study that provided the result in a form most suitable for analysis (the outcome was “Physical activity” and one of the studies reported a large number of continuous (e.g., number of blocks walked/week) and dichotomous (e.g., proportion climbing no stairs/week) outcomes and one study summarised and dichotomised these into an “active or not” variable).

Eight studies used data from ACL survey, a multistage stratified area probability sample of persons 24 years of age or older who lived in the continental United States. One of these studies was not used in the data synthesis as it was judged to have a score of 5 on the risk of bias scale, four of these studies did not report data that enabled calculation of an effect size and standard error, so only one of these studies was used in the data synthesis.

Four studies used data from the AHEAD, a longitudinal survey of a United States nationally representative cohort of persons who were born in 1923 or before and were living in the community other than nursing homes at the time of the baseline interview in 1993. One of these studies was not used in the data synthesis as it was judged to have a score of 5 on the risk of bias scale, three of these studies did not report data that enabled calculation of an effect size and standard error, so none of these studies were used in the data synthesis.

Two studies used data from the PATH Through Life Project, a population based study of Australian adults. Both studies were judged to have a score of 5 on the risk of bias scale and were not included in the data synthesis.

Six studies (all by the same author) used data collected among older adults living in Nara Prefecture, Japan. Three of these studies were not used in the data synthesis as they were judged to have a score of 5 on the risk of bias scale, and among the three remaining studies two reported the same outcome, thus two studies were used in the data synthesis.

Two studies used data from the TLSA, a nationally representative cohort sample of adults who were 60 years of age and older. Both studies were judged to have a score of 5 on the risk of bias scale and were not included in the data synthesis.

Two studies used the SHARE, which is a multidisciplinary and cross‐national panel database of micro data on health, socio‐economic status and social and family networks of individuals aged 50 or older. The first wave of SHARE data (2004/2005) was from nationally representative samples drawn from population registries of noninstitutionalised populations aged 50 years and older in 11 European countries (Denmark, Sweden, Austria, France, Germany, Switzerland, Belgium, the Netherlands, Spain, Italy and Greece) and Israel. Currently there are 27 European countries (Austria, Germany, Sweden, Spain, Italy, France, Denmark, Greece, Switzerland, Belgium, Czech Republic, Poland, Ireland, Luxembourg, Hungary, Portugal, Slovenia, Croatia, Bulgaria, Cyprus, Finland, Latvia, Lithuania, Malta, Romania, Slovakia and Estonia) and Israel participating. Both studies were judged to have a score of 5 on the risk of bias scale and were not included in the data synthesis.

Two studies used the ELSA data. ELSA is a multidisciplinary study that contains detailed information on the health, economic and social circumstances of a representative sample in England aged 50 and over. The first wave of data was collected in 2002. One of the studies was judged to have a score of 5 on the risk of bias scale and was not included in the data synthesis, the other study did not report data that enabled calculation of an effect size and standard error, so none of these studies were used in the data synthesis.

Two studies used data from the WLS, a study that has followed a random sample of male and female Wisconsin high school graduates since their graduation in 1957. One of the studies was judged to have a score of 5 on the risk of bias scale and was not included in the data synthesis, the other study was used in the data synthesis.

Two studies used the LSOA data. LSOA is a (United States) nationally representative sample of noninstitutionalized persons aged 70 years and older. They were first interviewed in 1984 and follow up was conducted in 1986, 1988 and 1990. In addition one study used The Second Longitudinal Study of Aging (LSOA II) which is a new cohort of noninstitutionalized persons aged 70 years and older, first interviewed in 1994 with two follow up interviews, conducted in 1997–1998 and 1999–2000. One of the studies was judged to have a score of 5 on the risk of bias scale and was not included in the data synthesis, one study did not report data that enabled calculation of an effect size and standard error, so one of these studies was used in the data synthesis.

Two studies used the MSSA data. MSSA is a U.S. longitudinal cohort of high‐functioning older adults from three sites of the Established Populations for the Epidemiologic Study of the Elderly. Baseline data collection was conducted in 1988 and follow‐up conducted in 1991 and 1995. None of these studies reported data that enabled calculation of an effect size and standard error, so none were used in the data synthesis.

Two studies (by the same team of authors) used data of 5401 community‐dwelling older adults aged 65 years or older who were living in Kami town, Hyogo prefecture, Japan. One of the studies was judged to have a score of 5 on the risk of bias scale and was not included in the data synthesis, the other study was used in the data synthesis.

In Table 1 we show the total number of studies, that met the inclusion criteria for this review. The first column shows the total number of studies grouped by country of origin. The second column shows the number of these studies that did not provide enough data to calculate an effect estimate. The third column gives the number of studies that were coded with very high risk of bias. The fourth column gives the number of studies that were excluded from the data synthesis due to overlapping samples. The last column gives the total number of studies used in the data synthesis.

**Table 1:** Number of included studies by country

		Reduction due to			
Country	Total	Missing data	Too high risk of bias	Used same data sets	Used in data synthesis
Asian contries^a^	1		1		0
Australia	5		4		1
Brazil	1		1		0
Canada	2	1	1		0
China	1		1		0
European countries and Israel^b^	2		2		0
India	1		1		0
Ireland	1				1
Israel	2				2
Japan	11		4	1	6
Korea	3		2		1
New Zealand	1		1		0
Sweden	1		1		0
Taiwan	3		3		0
UK	2	1	1		0
United States	53	16	23	1	13
Total	90	18	46	2	24

*Note*: The reduction due to too high risk of bias preceded the reduction due to using same data set.

a The countries included in this study are Hong Kong, Japan, Singapore, South Korea and Taiwan.

b The countries included in these two studies are Denmark, Sweden, Austria, France, Germany, Switzerland, Belgium, the Netherlands, Spain, Italy, Greece, Czech Republic, Ireland, Israel, Poland, Luxembourg, Hungary, Portugal, Slovenia and Estonia.

Forty six studies were judged to have a score of 5 on the risk of bias scale for either the confounding item (43), for the incomplete data item (3), for the other bias item (29) or for the selective reporting item (2) (see a Supporting Information document for the detailed risk of bias assessments). Several of the studies were judged to have a score of 5 on one or more of the risk of bias items. In accordance with the protocol, we excluded studies scoring 5 on any of the risk of bias items from the data synthesis on the basis that they would be more likely to mislead than inform. Eighteen studies did not provide enough information enabling us to calculate an effects size and standard error or did not provide results in a form enabling us to use it in the data synthesis. All studies are listed in section *Characteristics of included studies* along with the reason if the study is not used in the data synthesis.

The main characteristics of the 24 studies used in the data synthesis are shown in Table 2 below.

**Table 2:** Characteristeristic of studies used in data synthesis

Characteristic (number of studies reporting)		
Baseline year (20)	Average (*SD*)	2001 (9.5)
	Range	1984–2014
Follow up years (24)	Average (*SD*)	5 (5.0)
	Range	0–25
Number of participants, volunteers (20)	Average (*SD*)	2,369 (6,093)
	Range	15–27,131
Number of participants, control (20)	Average (*SD*)	13,581 (49,235)
	Range	13–217.297
Number of participants, total (24)	Average (*SD*)	14,566 (49,097)
	Range	28–244.428
Percent female, volunteers (9)	Average (*SD*)	61 (15.4)
	Range	45.3–92.0
Percent female, total (13)	Average (*SD*)	57 (4.3)
	Range	51.6–67.0
Mean age,^a^ volunteers (5)	Average (*SD*)	76 (6.1)
	Range	70.5–83
Mean age,^a^ total (10)	Average (*SD*)	70.0 (4.2)
	Range	63–76.8
Mean years of education, volunteers (2)	Average (*SD*)	13 (0.6)
	Range	12.16–13
Percent with 13+ years of education, volunteers (5)	Average (*SD*)	30.6 (8.9)
	Range	23.7–43.7
Mean years of education, total (5)	Average (*SD*)	12 (1.4)
	Range	10.1–13.13
Percent with 13+ years of education, total (5)	Average (*SD*)	25.5 (12.2)
	Range	9.2–38.6
Type of voluntary work (1)	Education	100%
Hours per week (3)	Range	2–15
	Average (*SD*)	6.9 (7.1)

a All studies reported a minimum age or the percent above 65 years of age.

The baseline time period (the year the voluntary work that was analysed was measured) spanned by the included studies is 30 years, from 1984 to 2014 and on average the baseline year was 2001. On average the number of follow up years was 5, although with great variation from 0 to 25 years. The average number of volunteers analysed (not reported in four studies) was 2,369, ranging from 15 to 27,131 and the average number of controls was 13,581, ranging from 13 to 217.297. In total the average number of participants analysed was 14,566, ranging from 28 to 244.428. Not all studies reported an average age of the participants but among those reporting an average age of volunteers the average was 76 years and among those studies only reporting an average age of all participants (volunteers and controls combined) the average was 70 years. Likewise, a limited number of studies reported other characteristics of study participants. On average females constituted a little more than half of volunteers, 61% (reported in nine studies) and 57% of volunteers and controls combined (reported in 13 studies). The mean number of years of education was 13 among volunteers (reported in only two studies) and 12 among all study participants (reported in five studies). Another five studies reported that 30.6% of the volunteers had 13 or more years of education and yet another five studies reported that among volunteers and controls combined 25.5% had 13 or more years of education. Only one study reported the type of voluntary work (education) and three reported on the average number of hours per week.

#### 5.1.3 Excluded studies

In addition to the 90 studies that met the inclusion criteria for this review, 35 studies at first sight appeared relevant but did not meet our criteria for inclusion. The studies and reasons for exclusion are given in section *Characteristics of excluded studies*.

#### 5.1.4 Studies awaiting classification

Seventeen studies have been identified as potentially eligible but have not been incorporated into the review. The main reason is that there were insufficient information to determine whether the study participants meet the age eligibility criteria of the review. Despite attempts to contact the author(s) of all studies with insufficient information (we could not locate a valid e‐mail address for the authors of five of the studies) we are still awaiting answers with regard to 10 studies. Further two studies were unobtainable despite efforts to locate them through libraries and searches on the internet. The references to these studies are provided in section *References to studies awaiting classification*.

### 5.2 Risk of bias in included studies

The risk of bias coding for each of the 90 studies is shown in a Supporting Information document.

Three studies reported on the same RCT but none of the studies reported the method of randomisation and was judged unclear on the sequence generation and allocation concealment items. A further three studies used a randomised design of which two reported using an appropriate method of randomisation and were rated low risk of bias on the sequence generation and allocation concealment items. The remaining RCT did not report the method of randomisation and was judged unclear on the sequence generation and allocation concealment items. One of the RCTs had a published a priori protocol and a priori analysis plan.

The remaining 84 studies used nonrandomised designs, they were all judged to have a high risk of bias on the sequence generation item and the allocation concealment item. None of the nonrandomised studies had an a priori protocol or an a priori analysis plan.

A summary of the risk of bias associated with blinding, incomplete data, selective reporting, other bias and confounding is shown in Table 3. Due to the nature of the intervention it was not possible to blind the participants and the majority of studies were given a score on 4 on the blinding item. If, however, the study reported on an objective outcome (as, e.g., mortality or long‐term care certificates in Japan) or outcome assessors were blinded, the study was given a score of 3 (20 studies) and one study applied further procedures to blind participants and members of the study team and was given a score of 2. Almost a third (28) of the studies did not report any information concerning attrition or missing data and could therefore not be rated on the incomplete data item. Three studies were given a score of 5 on the incomplete data item, corresponding to a risk of bias so high that the findings should not be considered in the data synthesis. A few studies had selective reporting issues, although two studies were given a score of 5 on the selective reporting item, corresponding to a risk of bias so high that the findings should not be considered in the data synthesis. Almost a third (29) of the studies were given a score of 5 on the other bias item, corresponding to a risk of bias so high that the findings should not be considered in the data synthesis. The confounding item was only judged for the 84 nonrandomised studies, of these 43 were given a score of 5, corresponding to a risk of bias so high that the findings should not be considered in the data synthesis and 29 studies did not either show or discuss imbalances on any of the confounders and were thus rated unclear on the confounding item.

**Table 3:** Summary risk of bias score

	Judgement								
RoB item	High	Low	Unclear	1	2	3	4	5	Number of studies
Sequence generation	84	2	4						90
Allocation concealment	84	2	4						90
Blinding			0	0	1	20	69	0	90
Incomplete data			28	9	18	17	15	3	90
Selective reporting			0	82	1	1	4	2	90
Other bias			6	41	8	3	3	29	90
Confounding			29	0	1	6	5	43	84

Some of the studies were given a score of 5 on several items. The total number of studies with a risk of bias so high that the findings should not be considered in the data synthesis was 46, corresponding to a little more than half of the studies. Most of the studies (30) with a risk of bias so high that the findings should not be considered in the data synthesis were not able to identify a causal effect, as all variables used (treatment, confounders and outcome) were measured at the same time. Others (13) failed to establish a comparison group that was balanced on important confounders and also controlled for outcomes that occur posttreatment, that is, they were bad controls. The remaining three studies scored 5 on the Incomplete data item (2) and Selective reporting item (1).

### 5.3 Synthesis of results

#### 5.3.1 Physical outcomes

##### Mortality

In order to carry out a meta‐analysis, every study must have a comparable effect size. Ten studies analysed the effect of voluntary work on mortality, however, eight studies reported a hazard ratio and two studies reported an odds ratio. We analysed these two types of effect sizes separately.

**Figure 2:**
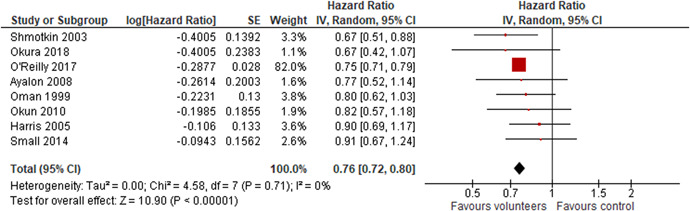
Mortality, hazard ratio

A hazard ratio <1 indicates that the treated, the volunteers is favoured. That is, the conditional mortality rate is lower for volunteers. All reported results indicated an effect favouring the volunteers. The random effects weighted mean hazard ratio was 0.76 (95% CI, 0.72–0.80) and statistically significant. The Forest plot is displayed in Figure 2. There is no heterogeneity between the studies; the estimated *τ* ^2^ is 0.00, *Q* = 4.58, df = 7 and *I* ^2^ is 0% as displayed in Figure 2.

**Figure 3:**
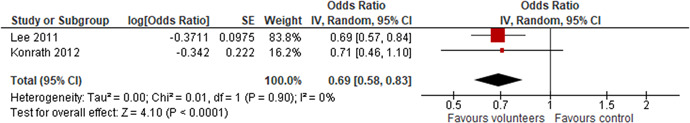
Mortality, odds ratio

An odds ratio <1 indicates that the treated, the volunteers is favoured. That is, the odds of dying is lower for volunteers. Both reported results indicated an effect favouring the volunteers. The random effects weighted mean odds ratio was 0.69 (95% CI, 0.58–0.83) and statistically significant. The Forest plot is displayed in Figure 3. There is no heterogeneity between the studies; the estimated *τ* ^2^ is 0.00, *Q* = 0.01, df = 1 and *I* ^2^ is 0% as displayed in Figure 3.

#### 5.3.2 Other physical outcomes

Three studies analysed the effect of voluntary work on incident functional disability, using a hazard ratio as effect measure.

**Figure 4:**
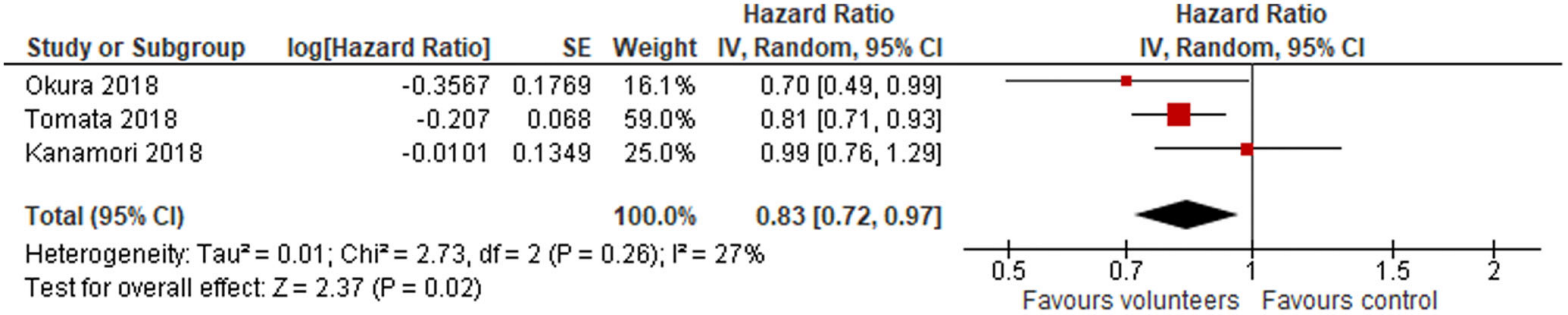
Incident functional disability

A hazard ratio <1 indicates that the volunteers is favoured. That is, the conditional incident functional disability rate is lower for volunteers. All reported results indicated an effect favouring the volunteers. The random effects weighted mean hazard ratio was 0.83 (95% CI, 0.72–0.97) and statistically significant. The Forest plot is displayed in Figure 4. There is a small amount of heterogeneity between the studies; the estimated *τ* ^2^ is 0.01, *Q* = 2.73, df = 2 and *I* ^2^ is 27% as displayed in Figure 4.

**Figure 5:**
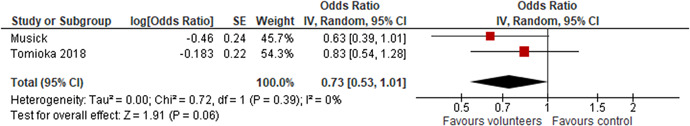
Decline in instrumental activities of daily living

Two studies analysed the effect of voluntary work on decline in IADL, using an odds ratio as effect measure. An odds ratio <1 indicates that the treated, the volunteers is favoured. That is, the odds of a decline in IADL is lower for volunteers. Both reported results indicated an effect favouring the volunteers. The random effects weighted mean odds ratio was 0.73 (95% CI, 0.53–1.01) and not statistically significant. The Forest plot is displayed in Figure 5. There is no heterogeneity between the studies; the estimated *τ* ^2^ is 0.00, *Q* = 0.72, df = 1 and *I* ^2^ is 0% as displayed in Figure 5.

**Figure 6:**
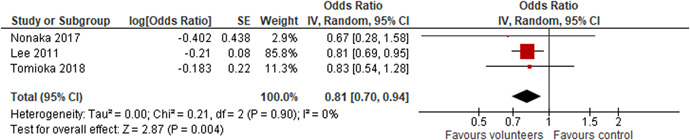
Maintenance of functional competence

Three studies analysed the effect of voluntary work on maintenance of functional competence, using an odds ratio as effect measure. An odds ratio <1 indicates that the treated, the volunteers is favoured. That is, the odds of not maintaining their functional competence is lower for volunteers. All reported results indicated an effect favouring the volunteers. The random effects weighted mean odds ratio was 0.81 (95% CI, 0.70–0.94) and statistically significant. The Forest plot is displayed in Figure 6. There is no heterogeneity between the studies; the estimated *τ* ^2^ is 0.00, *Q* = 0.21, df = 1 and *I* ^2^ is 0% as displayed in Figure 6.

In addition a number of outcomes were reported in a single study only. The outcomes were physical activity, cognitive impairment, incident CVD, functional impairment, doctor visits and nights in hospital. The effect sizes and 95% CIs are reported in section *Effect sizes*.

### 5.4 Mental health outcomes

Three studies analysed the effect of voluntary work on depression, and reported results that enabled the calculation of SMD and variance. The effect sizes are measured such that a positive effect size favours the volunteers, that is, when an effect size is *positive* the voluntary workers are better off than control groups of nonvolunteers, and when an effect size is *negative* the voluntary workers are worse off than control groups of nonvolunteers.

**Figure 7:**
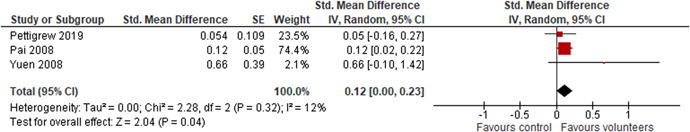
Depression

The random effects weighted SMD was 0.12 (95% CI, 0.00–0.23) and statistically significant. The Forest plot is displayed in Figure 7. There is a very small amount of heterogeneity between the studies; the estimated *τ* ^2^ is 0.00, *Q* = 2.28, df = 2 and *I* ^2^ is 12% as displayed in Figure 7.

In addition, a number of mental health outcomes were reported in a single study only. The outcomes were psychological well‐being, life satisfaction, self‐efficacy, quality of life, purpose in life, personal growth, self‐esteem and loneliness. The effect sizes and 95% CIs are reported in section *Effect sizes*.

### 5.5 Secondary outcomes

A number of secondary outcomes were reported in a single study only. The outcomes were functional limitations, self‐rated health, decline in cognitive performance and life satisfaction. The effect sizes and 95% CIs are reported in section *Effect sizes*.

### 5.6 Sensitivity analysis

We carried out a sensitivity analysis in the analysis of mortality based on hazard ratios only; as the number of studies included in the other meta analyses were too low (three studies as maximum).

Sensitivity analyses were planned to evaluate whether the pooled effect sizes were robust across study design and components of methodological quality.

No RCTs were included in the meta analysis on mortality, so the impact of study design could not be evaluated. For methodological quality, we carried out sensitivity analyses for the incomplete outcome data and confounding risk of bias components of the risk of bias checklists. We examined the robustness of our conclusions when we excluded studies with risk of bias score Unclear and 3 on the incomplete outcome data item and studies with risk of bias score Unclear and 4 on the confounding item.^7^

The results of the sensitivity are provided in Table 4 and displayed in Forest plots in section *Funnel Plots*.

**Table 4:** Sensitivity analysis

	Number of studies	Mean	95% CI	
Studies excluded	*k*	HR	Lower	Upper
	8	0.76	0.72	0.8
Incomplete outcome data score Unclear and 3	4	0.83	0.73	0.95
Confounding score Unclear and 4	4	0.85	0.73	0.98

*Note*: Exclusion of studies with score Unclear and 3 on the incomplete outcome data item and studies with score Unclear and 4 on the confounding risk of bias items. HRs with 95% CI.

Abbreviations: CI, confidence interval; HR, hazard ratios.

There were no appreciable changes in the results following removal of any of the studies.

In summary, the conclusion of the main synthesis do not change.

## 6 DISCUSSION

### 6.1 Summary of main results

This review focused on the effect of voluntary work on the physical and mental health of the volunteers.

#### 6.1.1 Physical outcomes

The available evidence does suggest that there is an effect on the mortality of volunteers, although the effect is small. We found a statistically significant negative effect of volunteering on mortality. The effects were measured by hazard ratios in eight studies and two studies reported odds ratios. In the context of hazard ratios (the ratio of two hazard rates), the hazard is the rate within a short time interval at which the participants die, conditional on staying alive. In other words, the probability of dying in that short time interval is the hazard rate. The weighted average effect (using the eight studies reporting hazard ratios) measured as a hazard ratio is 0.76, which translates into a decrease of approximately 24% in the hazard rate of death. The effect thus favoured the volunteers. The two studies that reported odds ratios of mortality supported this result. There was no heterogeneity between the studies in either of the meta analyses.

Interpretation of the result of a 24% decrease in the hazard rate of death would ideally involve a measure of the average hazard rates for the control group. However, none of the studies reported such rates.

The interpretation of a hazard ratio <1 is that a volunteer who is still alive by a certain time has a lower chance of dying at the next point in time compared to someone in the control group.

There is an alternative interpretation of the hazard ratio that may be intuitively easier to understand. The hazard ratio is equivalent to the odds that an individual in the group with the higher hazard reaches the endpoint (dies) first.

Stated another way, for any random pair of participants, one from the treatment group (the volunteers) and one from the control group, the hazard ratio is the odds that the time to death is shorter for the people from the treatment group than in the people from the control group. The probability of dying first (*p*) can easily be derived from the odds or hazard ratio (HR) of dying first, which is the probability of dying first divided by the probability of not dying first: HR = odds = *p*/(1 − *p*); *p* = HR/(1 + HR) (Spotswood et al., 2004). A hazard ratio of 0.76 therefore corresponds to a 43% chance of the volunteers dying first. Note this chance should be compared to a fifty‐fifty chance (50%) of dying first if the intervention had no effect. The lower and upper 95% CI corresponds to a 42–44% chance of the volunteers dying first.

For other physical outcomes, the evidence was inconclusive because too few studies contributed data.

#### 6.1.2 Mental health outcomes

The evidence was inconclusive because too few studies contributed data.

#### 6.1.3 Secondary outcomes

The evidence was inconclusive because too few studies contributed data.

### 6.2 Overall completeness and applicability of evidence

In this review, we included total of 24 studies in the data synthesis. This number is relatively low compared to the large number of studies (90) meeting the inclusion criteria. The reduction was caused by three different factors. Forty‐six studies were judged to have a very high risk of bias (5 on the scale) and, in accordance with the protocol, we excluded these from the data synthesis on the basis that they would be more likely to mislead than inform on the size of the effect of the intervention. Eighteen of the 90 studies did not report effect estimates or provide data that would allow the calculation of an effect size. Two studies could not be used because of overlapping data samples.

If all the 90 studies had provided an effect estimate with lower risk of bias, the final list of useable studies in the data synthesis would have been larger^8^ which, in turn, would have provided a more robust literature on which to base conclusions.

In total, 47 different populations from 16 countries were represented by the 90 studies meeting the inclusion criteria. The 24 studies used in the data synthesis covered only six countries (Australia, Ireland, Israel, Japan, Korea and United States). The more narrow geographical coverage may limit the applicability of the evidence.

We analysed all available physical and mental health outcomes. At most three studies were eligible for analysis of each of these outcomes with the exception of mortality. The small number of studies reporting other outcomes than mortality makes us reluctant to draw conclusions except concerning mortality.

We found no strong indication of publication bias.

### 6.3 Quality of the evidence

Overall the risk of bias in the majority of included studies was high. Forty six studies were judged to be at very high risk of bias. The risk of bias was examined using a tool for assessing risk of bias incorporating nonrandomised studies. We attempted to enhance the quality of the evidence in this review by excluding studies judged to be at very high risk of bias using this tool. We believe this process excluded those studies that are more likely to mislead than inform.

Furthermore, we performed a sensitivity analysis (where possible) to check whether the obtained result is robust across methodological quality. The overall conclusion did not change.

There was overall consistency in the direction of effects and there was no heterogeneity between studies.

### 6.4 Limitations and potential biases in the review process

We performed a comprehensive electronic database search, combined with grey literature searching, and hand searching of key journals. All citations were screened by two independent screeners from the review team (CAKI and ANDA), and one review author (TRF) assessed all included studies against inclusion criteria.

We believe that all the publicly available studies on the effect of voluntary work on the health of volunteers up to the censor date were identified during the review process. However, two references were not obtained in full text and 17 studies provided insufficient information to determine whether the study participants met the age eligibility criteria of the review. Despite attempts to contact the author(s) of all studies with insufficient information (we could not locate a valid e‐mail address for the authors of five of the studies) we are still awaiting answers with regard to 10 studies.

We were unable to comment on the possibility of publication bias as at most eight studies was included in the same meta‐analysis. Thus, we cannot rule out that there are still some missing studies.

We believe that there are no other potential biases in the review process as two members of the review team (CAKI and ANDA) independently coded the included studies. Any disagreements were resolved by discussion. Further, decisions about inclusion of studies were made by two members of the review team (CAKI and ANDA) and one review author (TRF). Assessment of study quality and numeric data extraction was made by one review author (TRF) and was checked by a second review author and two members of the review team (TF, ND, and AB).

### 6.5 Agreements and disagreements with other studies or reviews

We identified seven systematic reviews in the area of voluntary work and the health of older volunteers. Four of these offers only a narrative description of the studies, one an unweighted average of correlations, one reported effect sizes but did not pool them and one conducted a meta‐analysis that compare to our systematic review although it solely considered mortality.

Cattan et al. (2011) provides a comprehensive review of current knowledge (at that time, i.e., articles published between 2005 and 2011) regarding the role of volunteering in improving older (age not specified) people's quality of life. Studies that only analysed the impact on mortality or risk of disease were excluded. All study designs including descriptive and qualitative studies were eligible. Their search was systematic but they only offer a narrative description of the 22 included studies. The authors conclude that: “Although there are indications that volunteering may help to maintain and possibly improve some older adults' quality of life there are still major gaps in our knowledge regarding who actually benefits, the social and cultural context of volunteering and its role in reducing health and social inequalities “ (p. 331).

Bonsdorff von and Rantanen (2011) performed a systematic search (until November 30, 2009) of studies of volunteer work on personal well‐being: physical health, mental health and psychosocial resources. To be included, the study had to be published in a peer‐reviewed journal, written in English and conducted in western countries, participants were at least 60 years of age. Sixteen studies were included and a narrative review was provided. The conclusion offered by the authors was: “Volunteering in old age predicted better self‐rated health, functioning, physical activity and life satisfaction, as well as decreased depression and mortality. However, it did not decrease the risk of chronic diseases or nursing home admission in old age” (p. 167).

Milbourn et al. (2018) performed a systematic search of studies that assessed the relationship between time spent volunteering and quality of life in adults aged ≥50 years. They searched up to December 2017 and included only English language peer‐reviewed article published after January 2000. Eight studies were included (and one was excluded due to too low quality). The authors provided a narrative analysis of primary study results and concluded “Volunteering is identified as an important occupation for adults over the age of 50, although the relationship between time spent volunteering and the impact on quality of life outcomes is still not fully understood” (p. 613).

Anderson et al. (2014) performed a systematic search of studies that assessed the relationship between volunteering and benefits to the volunteers in adults aged ≥50 years. Searches were performed up to April 2014 and only English language peer‐reviewed articles were eligible. Seventy three studies were included of which 17 were descriptive studies without a comparison group. A narrative analysis of primary study results was provided. The conclusion is that “volunteering is associated with reduced symptoms of depression, better self‐reported health, fewer functional limitations and lower mortality” (p. 1).

Wheeler et al. (1998) searched systematic for studies of older volunteers (age not specified) doing all kinds of volunteer work (formal as well as informal) from 1965 to “the present”. Twenty nine studies were included of which 13 were descriptive studies without a comparison group or a one group before‐after study. An unweighted average of correlations (Pearson's linear correlation coefficient) between volunteering and life satisfaction was provided and the authors concluded that “engagement in meaningful volunteer activities probably positively affects nearly all older volunteers” (p. 76).

Guiney and Machado (2018) performed a systematic search of studies that assessed the relationship between volunteering (predictor) and cognitive functioning in adults aged ≥55 years. They searched up to June 2017 and included only English language peer‐reviewed article. They included 15 studies and reported effect sizes from primary studies (various measures, Cohen's *d*, Hedges *g*, unspecified betas and correlation) but did not provide a meta‐analysis. The overall conclusion was that there is evidence that “supports the idea that volunteering can protect against cognitive aging with respect to global functioning and at least some specific cognitive domain” (p. 399).

Contrary to the above mentioned six reviews, the conclusion in our review concerning mental health and physical outcomes (other than mortality) is that the evidence was inconclusive because too few studies contributed data.

Okun et al. (2013) performed a systematic search of studies that assessed the relationship between volunteering and mortality of the volunteers in adults aged ≥55 years up to November 3, 2011. They included only English language peer‐reviewed articles and book‐chapters. Fourteen studies were included. They performed a meta‐analysis using eleven studies, examining the relation between organisational volunteering and risk of mortality among adults 55 years old and older using hazard ratios. The weighted mean of these hazard ratios was 0.76, favouring the volunteers, with a 95% CI of 0.69–0.84. A moderate amount of heterogeneity between effect sizes were found (*I* ^2^ = 59%, *τ* ^2^ = 0.01). The weighted average effect size reported in Okun et al. (2013) is thus comparable to ours but where Okun et al. (2013) finds there is a moderate amount of heterogeneity between the studies we find no heterogeneity between studies. An explanation to this discrepancy may be that the effect sizes used in Okun et al. (2013) is a mix of estimated hazard ratios and standard errors as reported in the primary studies and “conversion” of estimated odds ratios and relative risk ratios (estimated using multinomial logistic models). Neither OR's nor RR's takes into account the differences in time in which each person is at risk of experiencing the event and there is no way to convert neither an OR nor a RR to a HR. The conversion formula reported in Okun et al. (2013) is not correct and may seriously bias the estimate of the HR. Another, or a supplementary, explanation may be that the outcomes from two studies performing multinomial logistic regression models are included. They are converted into a HR using first a conversion from OR into RR and then from RR into HR. However, the outcome from a multinomial logistic regression model is not an OR but a RR; and further the alternative to death (using a multinomial model) is not just staying alive but staying alive *and* being in good health. In one of the studies, Luoh and Herzog (2002), staying alive and being in good health is measured by self‐rated overall health and ADL limitations and in the other study, Gruenewald et al. (2007), measured by the lack of increases in self‐reported mobility disability and onset of difficulty in performing ADL). Okun et al. (2013) performs a meta regression in order to investigate if a number of factors (journal impact factor, year of publication, minimum age of sample, percentage of sample deceased and percentage of sample volunteering) can explain the observed heterogeneity but does not succeed. The reason may very well be that these factors are not the source of the observed heterogeneity but rather the inclusion of studies measuring mortality versus staying alive *and* being in good health.

## 7 AUTHORS' CONCLUSIONS

### 7.1 Implications for practice and policy

The review aimed to examine effects on all types of physical and mental health outcomes. With the exception of mortality, there was insufficient evidence available. Nevertheless, we found evidence that voluntary work reduces the mortality hazard of the volunteers aged 65 and above and the evidence seems robust in the sense that we did not find any heterogeneity between the studies even though they represented participants from a number of different countries (Ireland, Israel, Japan and United States) and spanned time periods ranging from 1984 to 2013. We cannot comment on the variety of types of volunteer work; that is, the extent to which it was done in different organisational contexts such as religious organisations, educational organisations, health organisations political groups, sports clubs, cultural organisations, senior citizen groups or related organisations, as none of the studies reported the rates of participants engaging in work done for different kinds of organisations (one study reported that all participants engaged in educational work).

Although the intervention is not completely free; organisations need staff time dedicated to train, support and recognise volunteers which is not costless, it is probably less costly than most other interventions in the social welfare area, and could be prescribed to more older people. In fact as the intervention in contrary to only carrying a cost is a productive activity too contributing directly to community well‐being and has a positive effect on the volunteers it probably should be prescribed universally. However, due to the very nature of the intervention, it is voluntary and it cannot be prescribed, although it can be encouraged to take up. More people could probably be encouraged to take up voluntary work if the opportunity was immediately available.

Increasing the availability of opportunities through organisations that define the content of the volunteer work, produce plans, recruit the volunteers, educate them if necessary, and lead them is vital. Equally important is the visibility of the organisations through advertisement in newspapers, on the Internet and public appeals on television or radio.

In the United States, programmes such as Senior Corps Programs, are organised in local nonprofit organisations. Such programmes aims to integrate the aging population into voluntary work by providing the opportunity for older people to apply their life experience to meeting community needs. Such programmes could be more widespread also outside of the United States.

### 7.2 Implications for research

In this review we found evidence that voluntary work reduces the mortality hazard of the volunteers aged 65 and above. Concerning other physical and mental health outcomes, the evidence was inconclusive.

By excluding from the data synthesis studies judged to be at very high risk of bias this review aimed at enhancing the quality of the evidence on the effects of doing voluntary work on the volunteers. We believe this process excluded those studies that are more likely to mislead than inform on the true effect sizes. Overall the risk of bias in the studies included in the review was high. Forty six studies were judged to be at very high risk of bias, corresponding to a risk of bias so high that the findings should not be considered in the data synthesis, leaving only 24 studies to be meta analysed. In almost all of the studies excluded from the data synthesis (43) the concern of too high risk of bias was on the confounding item. Most of these studies (30) were not able to identify a causal effect, as all variables used (treatment, confounders and outcome) were measured at the same time. Others failed to establish a comparison group that was balanced on important confounders and also controlled for outcomes that occur posttreatment.

These considerations point to the need for future studies that more thoroughly make use of the existing longitudinal data in order to identify a causal effect on health outcomes other than mortality.

Further, many of the available studies (18) did not provide data that permitted the calculation of an effect size and standard error or did not provide results in a form enabling us to use it in the data synthesis. If effect sizes and standard errors of these studies had been available, valuable information about the effect of voluntary work on outcomes other than mortality may possibly have been provided.

These considerations point to the need for studies reporting detailed results that permit their inclusion in systematic reviews.

## 8 METHODS NOT IMPLEMENTED

We planned to investigate the following factors with the aim of explaining potential observed heterogeneity: study‐level summaries of participant characteristics (e.g., studies considering a specific gender or socioeconomic level or studies where separate effects for men/women or low/high socioeconomic status are available) and type of voluntary work (religious, educational, political, etc.).

However, there was no heterogeneity between studies in the meta‐analysis of mortality limiting the need for performing a moderator analysis. Further, there was not enough variation in the covariates, to perform a moderator analysis (multiple meta‐regression using the mixed model or single factor subgroup analyses).

In the remaining meta analyses, there was either no or only a low degree of heterogeneity between studies and further, the number of included studies (in a single meta‐analysis) was not sufficient, to perform moderator analyses (multiple meta‐regression using the mixed model or single factor subgroup analyses).

## ROLES AND RESPONSIBILITIES

- Content: A. S. and T. F.
- Systematic review methods: T. F.
- Statistical analysis: T. F. and T. F.
- Information retrieval: B. C. V. N.

## SOURCES OF SUPPORT

VIVE‐Campbell.

## DECLARATIONS OF INTEREST

None.

## PRELIMINARY TIMEFRAME

Approximate date for submission of the systematic review is 1 year after protocol approval

## PLANS FOR UPDATING THE REVIEW

Once completed, we plan to update the review with a frequency of 2 years. Trine Filges will be responsible. Adelmann (1993), Bunout et al. (2012)

## Supporting information

Supporting information

Supporting information

Supporting information

Supporting information

## Footnotes

1 Australia, Austria, Belgium, Canada, Chile, Czech Republic, Denmark, Estonia, Finland, France, Germany, Greece, Hungary, Iceland, Ireland, Israel, Italy, Japan, Korea, Luxembourg, Mexico, the Netherlands, New Zealand, Norway, Poland, Portugal, Slovak Republic, Slovenia, Spain, Sweden, Switzerland, Turkey, UK and United States.

2 For the purpose of this review, “Europe” is defined as the 28 European Union countries, namely: Belgium, France, Italy, Luxembourg, the Netherlands, Germany, Denmark, Ireland, UK, Greece, Spain, Portugal, Austria, Sweden, Finland, Malta, Cyprus, Slovenia, Estonia, Latvia, Lithuania, Poland, the Czech Republic, Slovakia, Hungary, Romania and Bulgaria.

3 See note 1.

4 Members of the review team at VIVE Campbell were: the research assistants Caroline Fromberg Kiehn (CAKI), Anton Dam (ANDA) Anja Bondebjerg (AB) and researcher Nina Thorup Dalgaard (ND).

5 This risk of bias model was introduced by Prof. Reeves at a workshop on risk of bias in nonrandomised studies at SFI Campbell The former VIVE Campbell), February 2011. The model is a further development of work carried out in the Cochrane Non‐Randomised Studies Method Group.

6 See next page for an explanation of the terms selection bias and confounding.

7 The remaining studies scored 3 on the confounding item.

8 Avoiding overlap of data samples, 30 additional studies could have been used in the data synthesis.
