# Supplementary material for: Voluntary work for the physical and mental health of older volunteers: A systematic review
Source: Campbell Syst Rev. 2020 Oct 23;16(4):e1124. doi: 10.1002/cl2.1124 (PMC8356337; doi:10.1002/cl2.1124)
Supplement: Supplementary file 1 — Supporting information [file CL2-16-e1124-s003.docx]

# Characteristics of studies

## Characteristics of included studies

| Study | Data used | Country | Used in meta-analysis/reason not used |
| --- | --- | --- | --- |
| Armstrong, 2018 | Canadian General Social Surveys (2003, 2005, 2008, 2010 and 2013) | Canada | Too high risk of bias |
| Ayalon, 2008 | Between the years 1997 and 1998, the Israeli Central Bureau of Statistics conducted a face-to-face national survey of Israelis aged 60 years and older | Israel | Used in data synthesis |
| Becchetti, 2018 | Survey of Health, Ageing and Retirement in Europe (SHARE), waves 1, 2, 4 and 5 | Austria, Germany, Sweden, Netherlands, Spain, Italy, France, Denmark, Greece, Switzerland, Belgium, Israel, Czech Republic, Poland, Ireland, Luxembourg, Hungary, Portugal, Slovenia, and Estonia. | Too high risk of bias |
| Burr, 2011 | 2004 and 2006 Health and Retirement Study (HRS) | USA | Too high risk of bias |
| Burr, 2016 | Health and Retirement Study (2006-2010) | USA | Too high risk of bias |
| Burr, 2018 | Health and Retirement Study (2004-2014) | USA | Used in data synthesis |
| Carlson, 2008 | The Experience Corps program in Baltimore MD. | USA | Too high risk of bias |
| Carr, 2018a | Health and Retirement Study (HRS). The 2006–2014 HRS survey waves. | USA | Used in data synthesis |
| Carr, 2018b | Health and Retirement Study (1996–2012) | USA | Used in data synthesis |
| Chiao, 2018 | Taiwan Longitudinal Study on Aging (TLSA, 1993–2007) | Taiwan | Too high risk of bias |
| Choi, 2007 | The 1998 and 2000 interview waves of the Health and Retirement Study | USA | Too high risk of bias |
| Choi, 2013 | The first two waves of data collected by the Survey of Health, Ageing and Retirement in Europe (SHARE), (2004/2005) and (2006/2007) | Denmark, Sweden, Austria, France, Germany, Switzerland, Belgium, the Netherlands, Spain, Italy, Greece, Israel, the Czech Republic, Poland and Ireland | Too high risk of bias |
| Choi, 2016 | Five waves (2000–2008) of the Health and Retirement Study | USA | No relevant effect size reported |
| Corrêa, 2018 | Participants in this study were people age 60 years or older who attended the FaMIdade programme at the Granbery Methodist Institute in the city of Juiz de Fora, Brazil | Brazil | Too high risk of bias |
| Fonda, 2001 | The Asset and Health Dynamics among the Oldest Old (AHEAD) (1993–1995) | USA | Too high risk of bias |
| Fried, 2004 | A pilot randomized trial in Baltimore, Maryland (the experience corps). From November 1999 to January 2000. | USA | Not used in data synthesis due to overlap of data set |
| George, 2011 | RCT: older adults in the Northeast Ohio community, and Judson Park (JP), an assisted living facility in Cleveland, OH, that is registered in the Eden Alternative | USA | Not enough information provided to calculate standard error |
| Greenfield, 2004 | The 1995 Midlife Development in the United States (MIDUS) | USA | Too high risk of bias |
| Griep, 2017 | The 2010, 2012 and 2014 waves of the Swedish Longitudinal Occupational Survey of Health (SLOSH) | Sweden | Too high risk of bias |
| Gruenewald, 2007 | The MacArthur Study of Successful Aging | USA | Multinomial model, cannot identify relevant effect size |
| Han, 2017 | Health and Retirement Study (2006-2010) | USA | Multinomial model, cannot identify relevant effect size |
| Hao, 2009 | The Data for this study were obtained from the Asset and Health Dynamics among the Oldest Old (AHEAD) survey | USA | No relevant effect size reported |
| Harris, 2005 | The Longitudinal Study of Aging (LSOA). American community-dwelling older people | USA | Used in data synthesis |
| Heo, 2016 | 2008 wave of the Health and Retirement Study. | USA | Too high risk of bias |
| Ho, 2017 | Senior residents of Pingtung County aged 65 or more | Taiwan | Too high risk of bias |
| Hong, 2009 | The Longitudinal Study on Aging (LSOA) II | USA | Too high risk of bias |
| Huang, 2018 | Hong Kong, Japan, Singapore, South Korea, and Taiwan. World Values Survey data set collected from 2011 to 2012 | Asian contries | Too high risk of bias |
| Hunter, n.d. | Volunteers offered their services at the Veterans Administration Hospital (VAH) in Miami, Florida | USA | Too high risk of bias |
| Infurna, 2016 | Health and Retirement Study (HRS) data from 1998 to 2012 | USA | No relevant effect size reported |
| Jiang, 2018 | Personality and Total Health (PATH) Through Life Project | Australia | Too high risk of bias |
| Johnson, 2014 | Health and Retirement Study (HRS). Waves 1998 through 2004 | USA | Too high risk of bias |
| Jung, 2010 | Data from the first (1988) and second (1991) waves of the MacArthur Study of Successful Aging | USA | Cannot calculate ES and SE |
| Kahana, 2013 | Data for this research are part of the long-term longitudinal study of successful aging conducted by the Elderly Care Research Center, Wave 2 and 5, Florida | USA | No relevant effect size reported |
| Kanamori, 2014 | Aichi Gerontological Evaluation Study (AGES) Cohort Study data. | Japan | Used in data synthesis |
| Kim, 2010 | Three waves of the Americans’ Changing Lives (ACL), (1986, 1989, 1994) | USA | No relevant effect size reported |
| Kim, 2013 | Korean elderly women living in urban areas from the 2004 survey The Living Profile and Welfare Service Needs of Elderly People in Korea | Korea | Too high risk of bias |
| Kim, 2014 | Two waves of the National Social Life, Health, and Aging Project (NSHAP), the first collected in 2005/2006 and the second collected in 2010/2011 | USA | Too high risk of bias |
| Kim, 2016 | 2006 wave of the Health and Retirement Study | USA | Used in data synthesis |
| Kim, 2017 | Korean National Longitudinal Study on Aging (KLoSA) | Korea | Used in data synthesis |
| Klinedinst, 2014 | Survey of residents of a continuing care retirement community | USA | Too high risk of bias |
| Konrath, 2012 | Wisconsin Longitudinal Study | USA | Used in data synthesis |
| Krägeloh, 2015 | 399 community-dwelling retirement-aged New Zealanders | New Zealand | Too high risk of bias |
| Lee, 2011 | Health and Retirement Study in 2000 and 2002 followed to 2006. | USA | Used in data synthesis |
| Lee, 2016 | Women aged 65 or older in 25 regions in Seoul, the capital of Korea, and 5 regions outside of Seoul. Data were collected for 20 months (March 2011 to October 2012) by the Center for Home Care Nursing, St. Mari’s Hospital at the Catholic University of Korea. | Korea | Too high risk of bias |
| Li, 2005 | Three-wave panel data from the Americans’ Changing Lives survey | USA | Too high risk of bias |
| Li, 2006 | ACL (Americas Changing Lives survey) | USA |  |
| Li, 2013a | The 2006 Sample Survey of the Aged Population in Urban/Rural China | China | Too high risk of bias |
| Li, 2013b | Survey of Health and Living Status of the Elderly in Taiwan, a national longitudinal survey of older adults initially conducted in 1989, used 1999, 2003, and 2007 waves | Taiwan | Too high risk of bias |
| Lum, 2005 | Longitudinal data from the 1993 and 2000 panels of the Asset and Health Dynamics Among the Oldest Old Study (AHEAD) | USA | Multinomial model, cannot identify relevant effect size |
| Luoh, 2002 | Waves 3 and 4 of the Asset and Health Dynamics among the Oldest Old (AHEAD), 1998 and 2000 | USA | Multinomial model, cannot identify relevant effect size |
| McLaughlin, 1985 | Data from a representative sample of older Americans surveyed m 1974 | USA | Too high risk of bias |
| McMunn, 2009 | Wave 2 of the English Longitudinal Study of Ageing (ELSA), 2004 | UK | Too high risk of bias |
| Menec, 2003 | Aging in Manitoba (AIM) Study in Canada | Canada | Not enough information provided to calculate standard error |
| Moen, 2002 | (1994/95) and (1996/97) waves of the Cornell Retirement and Well-Being Study | USA | Too high risk of bias |
| Morrow-Howell, 2003 | Three waves of data from the Americans’ Changing Lives Study | USA | Too high risk of bias |
| Musick, 1999 | Three waves of the Americans’ Changing Lives (ACL), (1986, 1989, 1994) | USA | Not enough information provided to calculate standard error |
| Musick, 2003 | Three waves of data from the Americans’ Changing Lives data set (1986, 1989, 1994) | USA | Not enough information provided to calculate effect size and standard error |
| Nagarka, 2015 | Data were collected during June 2010 to June 2012 among individuals aged 60 and older from the city of Pune located in Western parts of India. | India | Too high risk of bias |
| Nazroo, 2012 | English Longitudinal Study of Ageing (ELSA) | UK | Cannot calculate effect size and standard error |
| Nonaka, 2017 | A prospective cohort of 1,320 community-dwelling older adults over 65 years of age, in a suburban city of Tokyo, Japan. | Japan | Used in data synthesis |
| Okun, 2010 | The Later Life Study of Social Exchanges (LLSSE) | USA | Used in data synthesis |
| Okura, 2017 | From April 2013, community-dwelling older adults aged 65 years or older, living in Kami town, Hyogo prefecture, Japan. | Japan | Too high risk of bias |
| Okura, 2018 | From April 2013, community-dwelling older adults aged 65 years or older, living in Kami town, Hyogo prefecture, Japan. | Japan | Used in data synthesis |
| Oman, 1999 | A cohort of 2025 community dwelling residents of Marin County, California, first examined in 1990–91. | USA | Used in data synthesis |
| O'Reilly, 2017 | Subgroup of The Northern Ireland Mortality Study (NIMS) | Ireland | Used in data synthesis |
| Otsuka, 2018 | Ohsaki Cohort 2006 Study | Japan | Used in data synthesis |
| Pai, 2009 | ACL Wave 1 and 2 | USA | Used in data synthesis |
| Pettigrew, 2019 | RCT from Australia | Australia | Used in data synthesis |
| Piliavin, 2007 | Wisconsin Longitudinal Study | USA | Too high risk of bias |
| Poulin, 2014 | Changing Lives of Older Couples (CLOC) from the Detroit Standard Metropolitan Statistical Area | USA | Too high risk of bias |
| Prolux, 2018 | Nine waves of data from the Health and Retirement Survey (HRS) (1998–2014) | USA | No relevant effect size reported |
| Sabin, 1993 | The Longitudinal Study on Aging (LSOA) 1984, 1986 and 1988 | USA | Cannot calculate effect size and standard error |
| Shmotkin, 2003 | The first wave of the Cross-Sectional and Longitudinal Aging Study (CALAS). Israeli Jewish population | Israel | Used in data synthesis |
| Small, 2014 | 278 community-dwelling older adults from a rural Midwestern county, Linn County, Iowa | USA | Used in data synthesis |
| Souza, 2011 | Elderly active in voluntary work in Porto Alegre (Voluntary Elderly) | Brazil | Too high risk of bias |
| Tackett, 2001 | For this study only (US) | USA | Too high risk of bias |
| Tan, 2006 | The Experience Corps program in Baltimore MD. Pilot 1999 to 2000 | USA | Used in data synthesis |
| Tang, 2009 | Three-wave panel data from the Americans’ Changing Lives survey | USA | Too high risk of bias |
| Tavares, 2013 | 2004 and 2006 Health and Retirement Study (HRS) | USA | Too high risk of bias |
| Tomioka, 2017a | Nara Prefecture, Japan. In January and February 2011, and 3 years later (2014). | Japan | Not used in data synthesis due to overlap of data set |
| Tomioka, 2017b | The Nara Data Health Survey of Baby Boomers, October 2015, residents who were born in the years 1945–1949 and covered by A City’s medical insurance system. | Japan | Too high risk of bias |
| Tomioka, 2017c | March 2014, residents in A City and B City in in Nara Prefecture, Japan. | Japan | Too high risk of bias |
| Tomioka, 2017d | The Nara Data Health Survey of Baby Boomers, October 2015, residents who were born in the years 1945–1949 and covered by A City’s medical insurance system. | Japan | Too high risk of bias |
| Tomioka, 2018a | March 2014 and a follow-up survey in November 2016. Data was obtained through a prospective cohort study conducted by A City in Nara Prefecture, Japan. | Japan | Used in data synthesis |
| Tomioka, 2018b | January to February, 2011 and follow up after 3 years (must be 2014). Nara Prefecture, Japan (A City and B Town). | Japan | Used in data synthesis |
| Walsh, 1986 | Conducted by Louis Harris and Associates, the 1981 study. Aging in the Eighties | USA | Too high risk of bias |
| Warburton, 2008 | Australian (Brisbane) case control study of 387 participants. | Australia | Too high risk of bias |
| Windsor, 2008 | PATH Through Life Project, a population based study of Australian adults, second wave of data collection undertaken in 2005–2006 | Australia | Too high risk of bias |
| Yu, 2018 | RAND Health and Retirement Study 2010 data collection wave | USA | Too high risk of bias |
| Yuen, 2008 | RCT: Residents from 5 long-term-care (LTC) facilities in Charleston County, South Carolina. | USA | Used in data synthesis |

## Characteristics of excluded studies

| Study | Exclusion reason |
| --- | --- |
| Adelmann, 1993 | No effect of volunteering reported (only continuous hours of volunteering) |
| Ahern, 2008 | No effect of volunteering reported (only importance of volunteering) |
| Aquino, 1996 | Recursive model, total effect on relevant outcome of volunteer work not provided |
| Bond, 1982 | Wrong age |
| Cheung, 2006 | Volunteer variable a composite measure of five items, some were irrelevant |
| Choi, 2016 | Wrong age |
| Chop, 2011 | Wrong age |
| Cutler, 1981 | Not about formal volunteer work |
| Dorfman, 1994 | No relevant outcome |
| Fried, 2014 | No results reported |
| Fujiwara, 2009 | No non-activity control; control group also do some volunteer work |
| Guo, 2018 | Not about formal volunteer work |
| Gupta, 2018 | Volunteer variable too inclusive and no effect of volunteering reported (only continuous (change in) hours of volunteering) |
| Hansen, 2018 | Wrong age* |
| Herrera, 2001 | Unclear what volunteer variable measures |
| Hidalgo, 2013 | Wrong age* |
| Hong, 2010 | Wrong age* |
| Hsu, 2007 | Volunteer variable too inclusive |
| Hughes, 2013 | Volunteer variable too inclusive |
| Iveniuk, 2016 | Volunteer variable too inclusive |
| Jirovec, 1999 | No control group |
| Martin, 2009 | Volunteer question answered by proxy informants |
| Martinez, 2006 | Control group contaminated by other volunteer work |
| McDonnall, 2011 | Volunteer variable too inclusive |
| Mellor, 2009 | Wrong age |
| Okun, 2011 | Wrong age |
| Parisi, 2014 | No non-activity control; control group referred to a usual volunteer activity control condition. Outcome not relevant (typical frequency of participation in various lifestyle activities (e.g., cooking, singing, gardening, listening to music, reading) |
| Parkinson, 2010 | Reverse causal relation examined (only) |
| Pilkington, 2012 | Wrong age* |
| Sneed, 2013 | Wrong age* |
| Vijay, 2016 | No non-activity control; control group referred to a usual volunteer activity control condition. Outcome not relevant (Walking activity) |
| Wahrendorf, 2010 | Wrong age |
| Walker, 1981 | No relevant outcome |
| Yasunaga, 2016 | No non-activity control; control group also do some volunteer work |
| Young, 1998 | Volunteer variable too inclusive |

*: Sufficient information on participants’ age provided by authors per e-mail.

# Data and analyses

## Effect sizes

*Other physical outcomes (HR, OR and RR less than 1 and a negative SMD indicates that the treated, the volunteers is favoured)*

| Outcome | Study | Measure | | Effect size (95% CI) |
| --- | --- | --- | --- | --- |
| Physical activity | Tan, 2006 | | Odds Ratio | 0.50 [0.22, 1.12] |
| Cognitive impairment | Kim, 2017 | | Odds Ratio | 0.70 [0.31, 1.58] |
| Incident cardiovascular disease | Burr, 2018 | | Hazard Ratio | 0.85 [0.71, 1.02] |
| Functional impairment | Pai, 2008 | | Std. Mean Difference | -0.13 [-0.23, -0.03] |
| Doctor visits | Kim, 2016 | | Rate Ratio | 1.03 [0.95, 1.12] |
| Nights in hospital | Kim, 2016 | | Rate Ratio | 0.73 [0.62, 0.86] |

*Other mental outcomes (a positive SMD indicates that the treated, the volunteers is favoured)*

| Outcome | Study | Measure | Effect size (95% CI) |
| --- | --- | --- | --- |
| Psychological well-being | Pettigrew, 2019 | Std. Mean Difference | 0.17 [-0.04, 0.39] |
| Life satisfaction | Yuen, 2008 | Std. Mean Difference | 0.63 [-0.13, 1.39] |
| Self-efficacy | Pettigrew, 2019 | Std. Mean Difference | 0.07 [-0.14, 0.29] |
| Quality of life | Pettigrew, 2019 | Std. Mean Difference | 0.06 [-0.16, 0.27] |
| Purpose in life | Pettigrew, 2019 | Std. Mean Difference | 0.13 [-0.08, 0.35] |
| Personal growth | Pettigrew, 2019 | Std. Mean Difference | 0.29 [0.08, 0.51] |
| Self-esteem | Pettigrew, 2019 | Std. Mean Difference | -0.03 [-0.24, 0.19] |
| Loneliness | Carr, 2018 | Std. Mean Difference | 0.02 [-0.10, 0.14] |

*Secondary outcomes (OR higher than one and a positive SMD indicates that the treated, the volunteers is favoured )*

| Outcome | Study | Measure | Effect size (95% CI) |
| --- | --- | --- | --- |
| Functional limitations | Carr, 2018 | Std. Mean Difference | 0.42 [0.36, 0.48] |
| Self-rated health | Yuen, 2008 | Std. Mean Difference | -0.77 [-1.53, -0.01] |
| Decline in Cognitive performance | Tomioka, 2018 | Odds Ratio | 1.26 [0.90, 1.77] |
| Life satisfaction | Pettigrew, 2019 | Std. Mean Difference | 0.00 [-0.21, 0.21] |

## Funnel plots

Funnel mortality


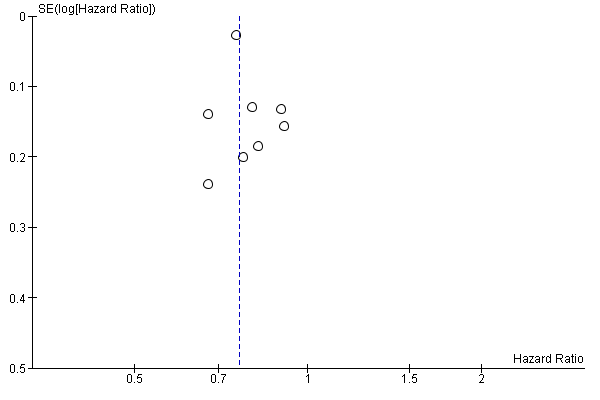


## Sensitivity analysis

# Appendices

## Search documentation

**Search results from bibliographic databases**

Below is a table showing the search results from each bibliographic database

| **Bibliographic Database** | **Search Results*** |
| --- | --- |
| SocIndex (EBSCO) | 1866 |
| PsycInfo (EBSCO) | 5570 |
| EconLit (EBSCO) | 3711 |
| Academic Search (EBSCO) | 4669 |
| Science Citation Index (Web of Science) | 1640****** |
| Social Science Citation Index (Web of Science) | 1640****** |
| MEDLINE (PubMed) | 1247 |
| Social Care Online | 397 |

*****Before removing duplicates.

******Combined for SCI & SSCI

**Search results from other resources and grey literature**

| **Resources** | **References screened*** |
| --- | --- |
| NBER Working Papers | 102 |
| Corporation for National and Community Service | 15 |
| OpenGrey | 183 |
| Royal Voluntary Service | 64 |
| UK Institute for Volunteering Research | 26 |
| Danish Institute for Voluntary Effort | 115 |
| Danish National Research Database | 599 |
| Volunteer Bénévoles/Canada | 1 |
| Google | ** |
| Google Scholar | 380 |

*Number indicates the total amount of references screened during the grey literature search on the resource.

**The first 100 results of searches were screened on Google. The total amount of references screened is to inexact to document exact.

### Search strings

**PsycINFO (EBSCO). Searched 3/12/2018.**

| **Search** | **Terms** | **Results** |
| --- | --- | --- |
| S11 | S3 OR S9 OR S10 | (5,570) |
| S10 | "Senior Corps" OR "Senior Companion Program" OR "Foster Grandparents Program" OR "Retired Senior and Volunteer Program" OR "Experience Corps" | (59) |
| S9 | S4 AND S7 AND S8 | (5,512) |
| S8 | AB (health* OR mortalit* OR mental* OR physic* OR psyc* OR social* OR wellbeing OR "quality of life" OR network* OR "life course*" OR gerontolo*) OR SU (“Health” OR ” Death and Dying” OR “Mortality Rate” OR “Mental Health” OR “Physical Health” OR “Physical Health Assessment”) | (4,526,994) |
| S7 | S5 OR S6 | (22,313) |
| S6 | SU “Volunteers" | (7,723) |
| S5 | AB (volunt* N8 (activi* OR engag* OR work* OR participa* OR involv* OR formal*)) | (16,974) |
| S4 | AB (old* OR age* OR agi* OR elder* OR senior* OR "late* life" OR retire*) | (1,059,584) |
| S3 | S1 AND S2 | (732) |
| S2 | TI (volunt*) | (10,998) |
| S1 | TI (old* OR age* OR agi* OR elder* OR senior* OR "late* life" OR retire*) | (192,533) |

**SocIndex (EBSCO). Searched 28/11/2018.**

| **Search** | **Terms** | **Results** |
| --- | --- | --- |
| S11 | S3 OR S9 OR S10 | (1,866) |
| S10 | "Senior Corps" OR "Senior Companion Program" OR "Foster Grandparents Program" OR "Retired Senior and Volunteer Program" OR "Experience Corps" | (37) |
| S9 | S4 AND S7 AND S8 | (1,683) |
| S8 | AB (health* OR mortalit* OR mental* OR physic* OR psyc* OR social* OR wellbeing OR "quality of life" OR network* OR "life course*" OR gerontolo*) | (850,615) |
| S7 | S5 OR S6 | (8,916) |
| S6 | SU “volunteer service” | (2,296) |
| S5 | AB (volunt* N8 (activi* OR engag* OR work* OR participa* OR involv* OR formal*)) | (7,616) |
| S4 | AB (old* OR age* OR agi* OR elder* OR senior* OR "late* life" OR retire*) | (334,414) |
| S3 | S1 AND S2 | (467) |
| S2 | TI (volunt*) | (5,514) |
| S1 | TI (old* OR age* OR agi* OR elder* OR senior* OR "late* life" OR retire*) | (82,777) |

**Academic Search (EBSCO). 03/12/2018.**

| **Search** | **Terms** | **Results** |
| --- | --- | --- |
| S11 | S3 OR S9 OR S10 | (4,669) |
| S10 | "Senior Corps" OR "Senior Companion Program" OR "Foster Grandparents Program" OR "Retired Senior and Volunteer Program" OR "Experience Corps" | (105) |
| S9 | S4 AND S7 AND S8 | (5,042) |
| S8 | AB (health* OR mortalit* OR mental* OR physic* OR psyc* OR social* OR wellbeing OR "quality of life" OR network* OR "life course*" OR gerontolo*) | (4,947,804) |
| S7 | S5 OR S6 | (33,131) |
| S6 | SU “volunteer service” | (8,168) |
| S5 | AB (volunt* N8 (activi* OR engag* OR work* OR participa* OR involv* OR formal*)) | (27,631) |
| S4 | AB (old* OR age* OR agi* OR elder* OR senior* OR "late* life" OR retire*) | (2,984,610) |
| S3 | S1 AND S2 | (1,030) |
| S2 | TI (volunt*) | (23,572) |
| S1 | TI (old* OR age* OR agi* OR elder* OR senior* OR "late* life" OR retire*) | (590,287) |

**EconLit (EBSCO). Searched 6/11/2018.**

| **Search** | **Terms** | **Results** |
| --- | --- | --- |
| S8 | S6 OR S7 | (3,711) |
| S7 | S1 AND S5 | (4,021) |
| S6 | "Senior Corps" OR "Senior Companion Program" OR "Foster Grandparents Program" OR "Retired Senior and Volunteer Program" OR "Experience Corps" | (2) |
| S5 | S2 OR S3 | (357,468) |
| S3 | AB (health* OR mortalit* OR mental* OR physic* OR psych* OR social* OR wellbeing OR "quality of life") | (160,628) |
| S2 | TI (health* OR mortalit* OR mental* OR physic* OR psych* OR social* OR wellbeing OR "quality of life") | (78,685) |
| S1 | TI volunt* OR AB volunt* | (10,059) |

**Social Science Citation Index & Science Citation Index (Web of Science). Searched 03/12/2018.**

| **Search** | **Terms** | **Results** |
| --- | --- | --- |
| # 5 | # 3 OR # 4 | 1,610 |
| # 4 | TS=("Senior Corps" OR "Senior Companion Program" OR "Foster Grandparents Program" OR "Retired Senior and Volunteer Program" OR "Experience Corps") | 122 |
| # 3 | #2 AND #1 | 1,601 |
| # 2 | TS=(health* OR mortalit* OR mental* OR physic* OR psyc* OR social* OR wellbeing OR "quality of life" OR network* OR "life course*" OR gerontolo*) | 8,063,138 |
| # 1 | TS=(Volunt* NEAR/8 (old* OR age* OR agi* OR elder* OR senior* OR "late* life" OR retire*) NEAR/8 (activi* OR engag* OR work* OR participa* OR involv*)) | 4,610 |

**MEDLINE (PubMed). Searched 07/12/2018.**

| **Search** | **Query** | **Items found** |
| --- | --- | --- |
| #7 | #5 OR #6 | 1247 |
| #6 | ("Senior Corps" OR "Senior Companion Program*" OR "Foster Grandparents Program*" OR "Retired Senior and Volunteer Program*" OR "Experience Corps") | 69 |
| #5 | #1 AND #2 AND #3 AND #4 | 1196 |
| #4 | (health*[Title/Abstract] OR mortalit*[Title/Abstract] OR mental*[Title/Abstract] OR physic*[Title/Abstract] OR psyc*[Title/Abstract] OR social*[Title/Abstract] OR wellbeing[Title/Abstract] OR "quality of life"[Title/Abstract] OR network*[Title/Abstract] OR "life course*"[Title/Abstract] OR gerontolo*[Title/Abstract]) | 4124353 |
| #3 | (old*[Title/Abstract] OR age*[Title/Abstract] OR agi*[Title/Abstract] OR elder*[Title/Abstract] OR senior*[Title/Abstract] OR "late* life"[Title/Abstract] OR retire*[Title/Abstract]) | 4334397 |
| #2 | (activi*[Title/Abstract] OR engag*[Title/Abstract] OR work*[Title/Abstract] OR participa*[Title/Abstract] OR involv*[Title/Abstract]) | 6296968 |
| #1 | volunt*[Title] | 33409 |

**Social Care Online. Searched 07/12/2018.**

| **Search** | **Terms** | **Results** |
| --- | --- | --- |
| #1 | TI= volunt* AND AB=(old* OR age* OR agi* OR elder* OR senior* OR "late* life" OR retire*) AND AB= (health* OR mortalit* OR mental* OR physic* OR psyc* OR social* OR wellbeing OR "quality of life" OR network* OR "life course*" OR gerontolo*) | 397 |

**Search strings on Google Scholar**

| **Search** | **Results** |
| --- | --- |
| allintitle: old volunteer | 83 |
| allintitle: old voluntary | 150 |
| allintitle: senior voluntary | 42 |
| allintitle: senior volunteer | 135 |
| allintitle: elder voluntary | 10 |
| allintitle: elder volunteer | 14 |

## Flow chart for literature search

**Screening**

**Included**

**Eligibility**

**Identification**

Records identified through database searching (*n* =19,100)

Additional records identified through other sources (*n* =1,509)

Records after duplicates (3,563) removed
(*n* =17,046)

Records screened (*n* =17,046)

Records excluded (*n* =16,674). Records not available (n=2).

Full-text articles assessed for eligibility (*n* =370)

Full-text articles excluded (*n* =265). Full-text articles awaiting classification (n=15).

References included (90 records)

References included in quantitative synthesis (meta-analysis) (*n* =24)

## First and second level screening

First level screening is on the basis of titles and abstracts. Second level is on the basis of full text

Reference id. No. :

Study id. No.:

Reviewers initials:

Source:

Year of publication:

Duration of study:

Country/countries of origin

Author

The study will be excluded if one or more of the answers to question 1-3 are ‘No’. If the answers to question 1 to 3 are ‘Yes’ or ‘Uncertain’, then the full text of the study will be retrieved for second level eligibility. All unanswered questions need to be posed again on the basis of the full text. If not enough information is available, or if the study is unclear, the author of the study will be contacted if possible.

**First level screening questions are based on titles and abstracts**

1. Are the participants’ individuals who do formal voluntary work?

Yes - include

No – if no then stop here and exclude

Uncertain - include

Question 1 guidance:

This includes all types of formal voluntary work described as on-going, planned, helping behaviour that intend to increase the well-being of strangers, offers no monetary compensation, and typically occurs within an organizational context (religious, educational or health organisations, political groups, senior citizen groups or related organizations). Informal ways of helping friends, neighbours, or relatives, such as running errands, providing transportation etc., which are typically motivated by an obligation to help intimate others, will be excluded.

1. Does the study include volunteers aged 65 and more?

Yes - include

No – if no then stop here and exclude

Uncertain - include

Question 2 guidance:

Studies where the majority of participants are aged 65 or over, or where results are shown for subgroups of participants aged 65 or over, will be included.

1. Is this study a primary quantitative study?

Yes - include

No – if no then stop here and exclude

Uncertain - include

Question 3 guidance:

We are only interested in primary quantitative studies, where the authors have analyzed the data. We are not interested in theoretical papers on the topic or surveys/reviews of studies of the topic. (This question may be difficult to answer on the base of titles and abstracts alone.)

**Second level screening questions based on full text**

1. Does the study estimate an effect, using a control group or using an estimated counterfactual?

Yes - include

No – if no then stop here and exclude

Uncertain - include

Question 4 guidance

E.g. 1) Randomised controlled trials including cluster randomisation and quasi randomised controlled study designs (i.e. participants are allocated by means such as alternate allocation, person’s birth date, the date of the week or month, case number or alphabetical order), 2) non randomised controlled study designs (i.e. quasi-experimental designs) such as controlled two group study designs or 3) study designs based on observational data, where the effect is estimated by statistical methods.

1. Does the study examine physical health (including mortality) or mental health?

Yes – include

No – if no then stop here and exclude

Uncertain – include

Question 5 guidance:

Examples of physical health outcomes include mortality, time until the onset of a serious disease (as for example a heart attack, stroke, cancer, arthritis), functional disability (measured by a standardized physical ability measure such as a difficulties in activities of daily living score (ADLs, see Katz et al., 1963)), or a difficulties in instrumental activities of daily living score (IADLs, see Lawton and Brody, 1969).

Examples of mental health outcomes include depression, anxiety and mental health-related disability measured by standardized psychological symptom measures such as the Center for Epidemiological Studies Depression Scale (CES-D), the Hopkins Symptom Checklist and the Medical Outcomes Study – Short Form.

## Data extraction

| **Authors** |
| --- |
| **Journal** |
| **Year** |
| **Country** |
| **Time period covered by data** |
| **Type of voluntary work** |
| **Participation characteristics (age, gender, education, ethnicity)** |
| **Hours of voluntary work** |
| **Type of data used (register, questionnaire, other (specify))** |
| **Sampling frequency** |
| **Time interval the outcome measure is based on (if different from sampling frequency)** |
| **Sample size (Treatment/control)** |

**Outcome measures**

Instructions: Please enter outcome measures in the order in which they are described in the report. Note that a single outcome measure can be completed by multiple sources and at multiple points in time (data from specific sources and time-points will be entered later).

| # | Outcome  & measure | Reliability & Validity | Format | Direction | Source | Pg# & notes |
| --- | --- | --- | --- | --- | --- | --- |
| 1 |  | Info from:  Other samples  This sample  Unclear  Info provided: | Dichotomy  Continuous  Time-to-event | High score or event is  Positive  Negative  Can’t tell | Questionnaire  Admin data  Other (specify)  Unclear |  |

* Repeat as needed

**DICHOTOMOUS OUTCOME DATA**

| OUTCOME | TIME POINT (s) (record exact time, there may be more than one, record them all) | SOURCE | VALID Ns | CASES | NON-CASES | STATISTICS | Pg. # & NOTES |
| --- | --- | --- | --- | --- | --- | --- | --- |
|  |  | Questionnaire  Admin data  Other (specify)  Unclear | Intervention | Intervention | Intervention | RR (risk ratio)  OR (odds ratio)  SE (standard error)  95% CI  DF  exact p value  Chi2  Other |  |
|  |  |  |  |  |  |  |  |
|  |  |  | Comparison | Comparison | Comparison |  |  |
|  |  |  |  |  |  |  |  |

Repeat as needed

**TIME-TO-EVENT OUTCOME DATA**

| OUTCOME | TIME POINT (s) (record exact time, there may be more than one, record them all) | SOURCE | Method of estimation |  |  | STATISTICS | Pg. # & NOTES |
| --- | --- | --- | --- | --- | --- | --- | --- |
|  |  | Questionnaire  Admin data  Other (specify)  Unclear | Non-parametric  Semi-parametric  Parametric |  |  | HR (hazard ratio)  SE (standard error)  95% CI  DF  exact p value  Chi2  Other |  |
|  |  |  |  |  |  |  |  |
|  |  |  |  |  |  |  |  |
|  |  |  |  |  |  |  |  |

Repeat as needed

**CONTINUOUS OUTCOME DATA**

| OUTCOME | TIME POINT (s) (record exact time, there may be more than one, record them all) | SOURCE  (specify) | VALID Ns | Means | SDs | STATISTICS | Pg. # & NOTES |
| --- | --- | --- | --- | --- | --- | --- | --- |
|  |  | Questionnaire  Admin data  Other (specify)  Unclear | Intervention | Intervention | Intervention | P, t or F  Df  ES  Covariates  Other |  |
|  |  |  |  |  |  |  |  |
|  |  |  | Comparison | Comparison | Comparison |  |  |
|  |  |  |  |  |  |  |  |

*Repeat as needed

## Assessment of risk of bias

**Risk of bias table**

| **Item** | **Judgement^a^** | **Description** (quote from paper, or describe key information) |
| --- | --- | --- |
| 1. Sequence generation |  |  |
| 2. Allocation concealment |  |  |
| 3. Confounding^b,c^ |  |  |
| 4. Blinding?^b^ |  |  |
| 5. Incomplete outcome data addressed?^b^ |  |  |
| 6. Free of selective reporting?^b^ |  |  |
| 7. Free of other bias? |  |  |
| *8. A priori* protocol?^d^ |  |  |
| *9. A priori* analysis plan?^e^ |  |  |

^a^ Some items on low/high risk/unclear scale (double-line border), some on 5 point scale/unclear (single line border), some on yes/no/unclear scale (dashed border). For all items, record “unclear” if inadequate reporting prevents a judgement being made.

^b^ For each outcome in the study.

^c^ This item is only used for NRCTs and NRSs. It is based on list of confounders considered important at the outset and defined in the protocol for the review (*assessment against worksheet*).

^d^ Did the researchers write a protocol defining the study population, intervention and comparator, primary and other outcomes, data collection methods, etc. in advance of starting the study?

^e^ Did the researchers have an analysis plan defining the primary and other outcomes, statistical methods, subgroup analyses, etc. in advance of starting the study?

**Risk of bias tool**

**Studies for which RoB tool is intended**

The risk of bias model was developed by Prof. Barnaby Reeves in association with the Cochrane Non-Randomised Studies Methods Group.^[[1]](#footnote-1)^ This model, an extension of the Cochrane Collaboration’s risk of bias tool, covers risk of bias in both randomised controlled trials (RCTs and QRCTs) and in non-randomised studies (NRCTs and NRSs).

The point of departure for the risk of bias model is the Cochrane Handbook for Systematic Reviews of interventions (Higgins & Green, 2008). The existing Cochrane risk of bias tool needs elaboration when assessing non-randomised studies because, for non-randomised studies, particular attention should be paid to selection bias / risk of confounding. Additional item on confounding is used only for non-randomised studies (NRCTs and NRSs) and is not used for randomised controlled trials (RCTs and QRCTs).

**Assessment of risk of bias**

Issues when using modified RoB tool to assess included non-randomised studies:

- Use existing principle: score judgment and provide information (preferably direct quote) to support judgment
- Additional item on confounding used only for non-randomised studies (NRCTs and NRSs).
- 5-point scale for some items (distinguish “unclear” from intermediate risk of bias).
- Keep in mind the general philosophy – assessment is not about whether researchers could have done better but about risk of bias; the assessment tool must be used in a standard way whatever the difficulty / circumstances of investigating the research question of interest and whatever the study design used.
- Anchors: “1/No/low risk” of bias should correspond to a high quality RCT. “5/high risk” of bias should correspond to a risk of bias that means the findings should not be considered (too risky, too much bias, more likely to mislead than inform)

1. Sequence generation

- Low/high/unclear RoB item
- Always high RoB (not random) for a non-randomised study
- Might argue that this item redundant for NRS since always high – but important to include in RoB table (‘level playing field’ argument)

2. Allocation concealment

- Low/high/unclear RoB item
- Potentially low RoB for a non-randomised study, e.g. quasi-randomised (so high RoB to sequence generation) but concealed (reviewer judges that the people making decisions about including participants didn’t know how allocation was being done, e.g. odd/even date of birth/hospital number)

3. RoB from confounding (additional item for NRCT and NRS; assess for each outcome)

- Assumes a pre-specified list of potential confounders defined in the protocol
- Low(1) / 2 / 3 / 4 / high(5) / unclear RoB item
- Judgment needs to factor in:
  - proportion of confounders (from pre-specified list) that were considered
  - whether most important confounders (from pre-specified list) were considered
  - resolution/precision with which confounders were measured
  - extent of imbalance between groups at baseline
  - care with which adjustment was done (typically a judgment about the statistical modeling carried out by authors)
- Low RoB requires that all important confounders are balanced at baseline (not primarily/not only a statistical judgment OR measured ‘well’ and ‘carefully’ controlled for in the analysis.

Assess against pre-specified worksheet. Reviewers will make a RoB judgment about each factor first and then ‘eyeball’ these for the judgment RoB table.

4. RoB from lack of blinding (assess for each outcome, as per existing RoB tool)

- Low(1) / 2 / 3 / 4 / high(5) / unclear RoB item
- Judgment needs to factor in:
  - nature of outcome (subjective / objective; source of information)
  - who was / was not blinded and the risk that those who were not blinded could introduce performance or detection bias
  - see Ch.8

5. RoB from incomplete outcome data (assess for each outcome, as per existing RoB tool)

- Low(1) / 2 / 3 / 4 / high(5) / unclear RoB item
- Judgment needs to factor in:
  - reasons for missing data
  - whether amount of missing data balanced across groups, with similar reasons
  - whether censoring is less than or equal to 25% and taken into account
  - see Ch.8

6. RoB from selective reporting (assess for each outcome, NB different to existing Ch.8 recommendation)

- Low(1) / 2 / 3 / 4 / high(5) /unclear RoB item
- Judgment needs to factor in:
  - existing RoB guidance on selective outcome reporting (see Ch.8)
  - also, extent to which analyses (and potentially other choices) could have been manipulated to bias the findings reported, e.g. choice of method of model fitting, potential confounders considered / included
  - look for evidence that there was a protocol in advance of doing any analysis / obtaining the data (difficult unless explicitly reported); NRS very different from RCTs. RCTs must have a protocol in advance of starting to recruit (for REC/IRB/other regulatory approval); NRS need not (especially older studies)
  - Hence, separate yes/no items asking reviewers whether they think the researchers had a pre-specified protocol and analysis plan.

7. RoB from other bias (assess for each outcome, NB different to existing Ch.8 recommendation)

- Low(1) / 2 / 3 / 4 / high(5) /unclear RoB item
- Judgment needs to factor in:
  - existing RoB guidance on other potential threats to validity (see Ch.8)
  - also, assess whether suitable cluster analysis is used (e.g. cluster summary statistics, robust standard errors, the use of the design effect to adjust standard errors, multilevel models and mixture models), if assignment of units to treatment is clustered

**Confounding Worksheet**

| **Assessment of how researchers dealt with confounding** |  |
| --- | --- |
| Method for *identifying* relevant confounders described by researchers: yes  no  If yes, describe the method used: |  |
| Relevant confounders described: yes  no  List confounders described on next page |  |
| Method used for controlling for confounding  At design stage (e.g. matching, regression discontinuity, instrument variable):  ………………………………………………..  ………………………………………………..  ………………………………………………..  At analysis stage (e.g. stratification, regression, difference-indifference):  ………………………………………………..  ………………………………………………..  ………………………………………………..  Describe confounders controlled for below |  |

**Confounders described by researchers**

Tick (yes[0]/no[1] judgment) if confounder considered by the researchers [Cons’d?]

Score (1[good precision] to 5[poor precision]) precision with which confounder measured

Score (1[balanced] to 5[major imbalance]) imbalance between groups

Score (1[very careful] to 5[not at all careful]) care with which adjustment for confounder was carried out

| **Confounder** | Considered | Precision | Imbalance | Adjustment |
| --- | --- | --- | --- | --- |
| Gender |  |  |  |  |
| Age |  |  |  |  |
| SES |  |  |  |  |
| Pre physical health |  |  |  |  |
| Pre mental health |  |  |  |  |
| Unobservables^[[2]](#footnote-2)^ |  | Irrelevant |  |  |
| Other: |  |  |  |  |

### User guide for unobservables

Selection bias is understood as systematic baseline differences between groups and can therefore compromise comparability between groups. Baseline differences can be observable (e.g. age and gender) and unobservable (to the researcher; e.g. motivation and ‘ability’). There is no single non-randomised study design that always solves the selection problem. Different designs solve the selection problem under different assumptions and require different types of data. Especially how different designs deal with selection on unobservables varies. The “right” method depends on the model generating participation, i.e. assumptions about the nature of the process by which participants are selected into a programme.

As there is no universal correct way to construct counterfactuals we will assess the extent to which the identifying assumptions (the assumption that makes it possible to identify the counterfactual) are explained and discussed (preferably the authors should make an effort to justify their choice of method). We will look for evidence that authors using e.g. (this is NOT an exhaustive list):

**Natural experiments:**

Discuss whether they face a truly random allocation of participants and that there is no change of behaviour in anticipation of e.g. policy rules.

**Instrument variable (IV):**

Explain and discuss the assumption that the instrument variable does not affect outcomes other than through their effect on participation.

**Matching (including propensity scores):**

Explain and discuss the assumption that there is no selection on unobservables, only selection on observables.

**(Multivariate, multiple) Regression:**

Explain and discuss the assumption that there is no selection on unobservables, only selection on observables. Further discuss the extent to which they compare comparable people.

**Regression Discontinuity (RD):**

Explain and discuss the assumption that there is a (strict!) RD treatment rule. It must not be changeable by the agent in an effort to obtain or avoid treatment. Continuity in the expected impact at the discontinuity is required.

**Difference-in-difference (Treatment-control-before-after):**

Explain and discuss the assumption that outcomes of participants and nonparticipants evolve over time in the same way.

1. This risk of bias model was introduced by Prof. Reeves at a workshop on risk of bias in non-randomised studies at SFI Campbell, February 2011. The model is a further development of work carried out in the Cochrane Non-Randomised Studies Method Group (NRSMG). [↑](#footnote-ref-1)
2. See user guide for unobservables [↑](#footnote-ref-2)
